# Fishborne zoonotic heterophyid infections: An update

**DOI:** 10.1016/j.fawpar.2017.09.001

**Published:** 2017-09-08

**Authors:** Jong-Yil Chai, Bong-Kwang Jung

**Affiliations:** Institute of Parasitic Diseases, Korea Association of Health Promotion, Seoul 07649, Republic of Korea

**Keywords:** Fishborne trematode, Zoonotic trematode, Heterophyid, *Metagonimus*, *Heterophyes*, *Haplorchis*

## Abstract

Fishborne heterophyid trematodes infecting humans are at least 29 species worldwide and belong to 13 genera. Its global burden is much more than 7 million infected people. They include *Metagonimus* (*M*. *yokogawai*, *M*. *takahashii*, *M*. *miyatai*, *M*. *minutus*, and *M*. *katsuradai*), *Heterophyes* (*H*. *heterophyes*, *H*. *nocens*, *H*. *dispar*, and *H*. *aequalis*), *Haplorchis* (*H*. *taichui*, *H*. *pumilio*, *H*. *yokogawai*, and *H*. *vanissimus*), *Pygidiopsis* (*P*. *summa* and *P*. *genata*), *Heterophyopsis* (*H*. *continua*), *Stellantchasmus* (*S*. *falcatus*), *Centrocestus* (*C*. *formosanus*, *C*. *armatus*, *C*. *cuspidatus*, and *C*. *kurokawai*), *Stictodora* (*S*. *fuscata* and *S*. *lari*), *Procerovum* (*P*. *varium* and *P*. *calderoni*), *Acanthotrema* (*A*. *felis*), *Apophallus* (*A*. *donicus*), *Ascocotyle* (*A*. *longa*), and *Cryptocotyle* (*C*. *lingua*). Human infections are scattered around the world but the major endemic areas are located in Southeast Asia. The source of human infection is ingestion of raw or improperly cooked fish. The pathogenicity, host-parasite relationships, and clinical manifestations in each species infection are poorly understood; these should be elucidated particularly in immunocompromised hosts. Problems exist in the differential diagnosis of these parasitic infections because of close morphological similarity of eggs in feces and unavailability of alternative methods such as serology. Molecular diagnostic techniques are promising but they are still at an infant stage. Praziquantel has been proved to be highly effective against most of the patients infected with heterophyid flukes. Epidemiological surveys and detection of human infections are required for better understanding of the geographical distribution and global burden of each heterophyid species. In this review, the most updated knowledge on the morphology, biology, epidemiology, pathogenesis and pathology, immunology, clinical manifestations, diagnosis and treatment, and prevention and control of fishborne zoonotic heterophyid infections is provided.

## Highlights

• Fishborne intestinal flukes are diverse and heterophyids are an important group.
• Thirteen heterophyid genera and 29 species are known to infect humans.
• *Metagonimus*, *Heterophyes*, and *Haplorchis* are the most important genera.
• Infections are significant due to the potential for morbidity and mortality.
• Epidemiological surveys and detection of human infections are urgently required.

## 1 Introduction

Foodborne zoonotic trematodes are highly diverse in terms of the species of parasites involved and the kinds and types of foods concerned. They can be largely divided into liver flukes, lung flukes, and intestinal flukes (Fürst et al., 2012, Chai, 2014a). The global burden of foodborne trematodes was estimated at about 50–60 million people (Fried et al., 2004, Fürst et al., 2012). However, this certainly is a far underestimate of the true number of infected people because of difficulty in case detection and diagnosis (Chai et al., 2009a).

Among them, intestinal flukes are the most neglected group despite a wide geographical distribution, a high prevalence in some endemic countries, and potentially significant morbidity and mortality. Intestinal trematodes are taxonomically diverse, and consist of more than 60 species worldwide (Chai et al., 2009a). They include heterophyids (including *Metagonimus yokogawai*, *Heterophyes nocens*, and *Haplorchis taichui*), echinostomes (including *Echinostoma revolutum*, *Echinostoma ilocanum*, *Echinochasmus japonicus*, *Artyfechinostomum malayanum*, and *Acanthoparyphium tyosenense*), fasciolids (*Fasciolopsis buski*), gymnophallids (*Gymnophalloides seoi*), microphallids (*Gynaecotyla squatarolae*), neodiplostomes/diplostomes/strigeids (*Neodiplostomum seoulense*), lecithodendriids (*Prosthodendrium molenkampi* and *Phaneropsolus bonnei*), and plagiorchiids (*Plagiorchis muris*) (Chai, 2007, Chai, 2014a, Chai et al., 2009a). Among them, the heterophyids have seldom been the subject of extensive reviews. Thus, in this article, the authors would like to review on the heterophyid group of intestinal flukes.

**Fig. 1:**
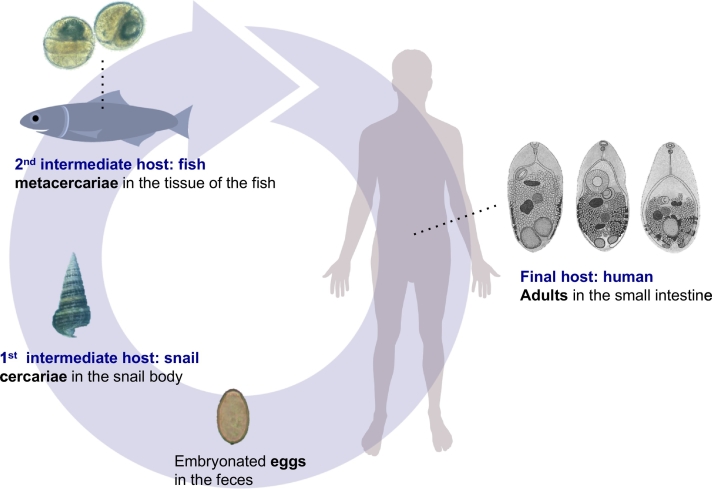
Common life cycle of fishborne zoonotic heterophyid trematodes. Human infection occurs when the infected fish are consumed raw or under improperly cooked conditions. Some images were adapted from Google.

Heterophyids (= family Heterophyidae Leiper, 1909) are a group of minute-sized (1–2 mm in length) trematodes infecting vertebrate animals, including mammals and birds (Yamaguti, 1958). At least 36 genera are known within this family (Pearson, 2008), and among them, 13 genera are known to be zoonotic (Chai, 2007); *Metagonimus*, *Heterophyes*, *Haplorchis*, *Pygidiopsis*, *Heterophyopsis*, *Stellantchasmus*, *Centrocestus*, *Stictodora*, *Procerovum*, *Acanthotrema*, *Apophallus*, *Ascocotyle*, and *Cryptocotyle*. They are exclusively fishborne and contracted to humans by ingesting raw or improperly cooked freshwater or brackish water fish (Chai, 2007, Chai, 2014a) (Fig. 1). Most of the infected people live in Asian countries, including Korea, China, Taiwan, Vietnam, Laos, Thailand, Malaysia, Indonesia, the Philippines, and India.

In view of the wide geographical distribution and the number of infected people around the world, *Metagonimus*, *Heterophyes*, and *Haplorchis* are the three most important genera (Chai, 2015). *Metagonimus* morphologically differs from *Heterophyes* and *Heterophyopsis* in that the former has a smaller, submedian-located ventral sucker and no genital sucker, whereas the latter two have a bigger and almost median-located ventral sucker and a prominent genital sucker (Chai, 2007, Yu and Chai, 2010, Yu and Chai, 2013). *Heterophyopsis* has an elongated body unlike *Heterophyes* (Chai, 2007). *Metagonimus* has two testes but *Haplorchis* and *Procerovum* have only one testis (Chai, 2015). *Haplorchis*, *Procerovum*, and *Stictodora* have prominent gonotyls armed with several to numerous rodlets, superimposed on the submedially located ventral sucker (Chai, 2015). *Pygidiopsis* has a small and median-located ventral sucker with the presence of a ventrogenital apparatus armed with 12–15 minute spines (Chai et al., 1986a). *Stellantchasmus* has a small, laterally deviated ventral sucker and an elongated sac-like seminal vesicle with a muscular expulsor at the opposite side of the ventral sucker (Yu and Chai, 2010, Yu and Chai, 2013, Chai, 2015). *Centrocestus* has minute circumoral spines on the oral sucker (Yu and Chai, 2010, Yu and Chai, 2013, Chai, 2015). *Stitodora* has a gonotyl armed with spines and a separate opening of the ejaculatory duct and metraterm, but lacks a muscular bulb in the genital atrium (Chai et al., 1988). *Acanthotrema* is similar to *Stictodora* but differs in having fewer than 12 sclerotizations on the ventral sucker; more than 12 spines in the latter (Sohn et al., 2003a, Sohn et al., 2003b).

The purpose of this review is to provide the most updated knowledge on the morphology, biology, epidemiology, pathogenesis and pathology, immunology, clinical manifestations, diagnosis and treatment, and prevention and control of fishborne zoonotic heterophyid infections. In this review, those species naturally occurring in humans, as well as those in which experimental human infection was successful, were included.

## 2 Morphology, biology, and epidemiology

### 2.1 Metagonimus

Flukes of *Metagonimus* are characterized by the presence of a small submedian and unarmed ventral sucker; they differ from *Heterophyes* in lacking a genital sucker and gonotyl and from *Haplorchis* in having two testes (Chai, 2007, Chai, 2017). The genus *Metagonimus* was erected with *M*. *yokogawai* as the type species (Katsurada, 1912, Yokogawa, 1913a, Ito, 1964a), and 8 more species (total 9 species) have been described to date. They include *M*. *ovatus* (Yokogawa, 1913b); *M*. *takahashii* (Suzuki, 1930), *M*. *minutus* (Katsuta, 1932a), *M*. *katsuradai* (Izumi, 1935), *M*. *otsurui* (Saito and Shimizu, 1968), *M*. *miyatai* (Saito et al., 1997), *M*. *hakubaensis* (Shimazu, 1999), and *M*. *suifunensis* (Shumenko et al., 2017) (Table 1). Among them, *M*. *yokogawai*, *M*. *takahashii*, and *M*. *miyatai* are the 3 major human-infecting species in Japan and Korea (Chai et al., 2005a, Chai et al., 2009a, Chai et al., 2015a). An experimental human infection was reported to be successful in *M*. *katsuradai* (Izumi, 1935), and *M*. *minutus* was included among the list of human-infecting species in Taiwan without literature background (Yu and Mott, 1994). Hence, 5 species, namely, *M*. *yokogawai*, *M*. *takahashii*, *M*. *miyatai*, *M*. *minutus*, and *M*. *katsuradai*, are regarded as zoonotic or potentially zoonotic species. It should be reminded that some of old literature on *M*. *yokogawai* is actually referring to *M*. *takahashii* or *M*. *miyatai*, and caution is required when reviewing *M*. *yokogawai* in strict sense (Chai et al., 2009a, Chai, 2015, Chai, 2017).

**Table 1:** Species of *Metagonimus* reported in the literature.

Species	Human infection	Country/region	First reporter
*Metagonimus yokogawai*	Yes	Korea, Japan, China, Taiwan, Russia, India, Europe	Katsurada (1912)
*Metagonimus takahashii*	Yes	Korea, Japan	Takahashi (1929a), Suzuki (1930)
*Metagonimus miyatai*	Yes	Korea, Japan	Katsurada (1912), Saito et al. (1997)
*Metagonimus minutus*	Yes^a^	Taiwan	Katsuta (1932a)
*Metagonimus katsuradai*	Yes^b^	Japan, Russia	Izumi (1935)
*Metagonimus ovatus*	No	Japan	Yokogawa (1913b)
*Metagonimus otsurui*	No	Japan	Saito and Shimizu (1968)
*Metagonimus hakubaensis*	No	Japan	Shimazu (1999)
*Metagonimus suifunensis*	No	Russia	Shumenko et al. (2017)

a Listed as a human-infecting species (Yu and Mott, 1994) without adequate documentation.

b Experimental human infection was reported.

#### 2.1.1 *Metagonimus yokogawai* (Katsurada, 1912) Katsurada, 1912

**Fig. 2:**
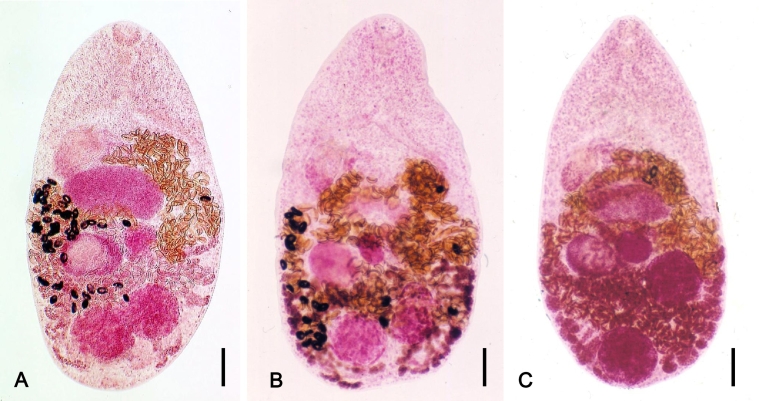
Morphology of *Metagonimus* spp. infecting humans. (A) *Metagonimus yokogawai*, (B) *Metagonimus takahashii*, and (C) *Metagonimus miyatai*. Note that *M*. *yokogawai* has two adjacent testes, whereas *M*. *takahashii* and *M*. *miyatai* have more or less two separated testes. The uterine tubule of *M*. *yokogawai* does not overlap with the anterior testis, but the tubules of *M*. *takahashii* and *M*. *miyatai* do overlap with the anterior testis. Vitelline follicles are distributed diffusely beyond the post-testicular areas in *M*. *takahashii* but not in *M*. *yokogawai* and *M*. *miyatai*. Scale bars; (A) = 100 μm, (B) = 100 μm, (C) = 100 μm.

The original description of this species is based on adult specimens recovered from an experimental dog fed the metacercariae in the sweetfish (*Plecoglossus altivelis*) from Taiwan (Katsurada, 1912, Shimazu and Kino, 2015). It is now known to distribute mainly in the Far Eastern countries (Chai, 2017). Its characteristic morphology include a minute body, a small laterally deviated ventral sucker with no ventrogenital apparatus and no genital sucker, a medially-located ovary, and two testes located almost side-by-side near the posterior end of body (Ito, 1964a, Chai et al., 2009a, Yu and Chai, 2013) (Fig. 2). *M*. *takahashii* and *M*. *miyatai* have two testes separated from each other (Chai et al., 2009a). In addition, the vitelline follicles of *M*. *yokogawai* extend in lateral fields from the level of the ovary down to the posterior end of the posterior testis, but not beyond the posterior testis (Saito et al., 1997, Chai, 2015). In *M*. *takahashii*, the vitelline follicles distribute abundantly beyond the posterior testis level (Chai et al., 2009a). The uterine tubules of *M*. *yokogawai* never overlap or cross over the middle portion of the anterior testis, whereas *M*. *takahashii* and *M*. *miyatai* have the uterine tubules which overlap the whole anterior testis (Chai et al., 1993a, Saito et al., 1997).

Freshwater snails (including *Semisulcospira libertina* and *S*. *coreana*) have been reported to be the molluscan intermediate host (Ito, 1964b, Cho et al., 1984). The most important fish host is the sweetfish (*Plecoglossus altivelis*) in Korea and Japan (Ito, 1964a, Chai and Lee, 2002, Sohn, 2009, Chai, 2007, Chai, 2015). The big-scaled redfin (*Tribolodon hokonensis*), Pacific redfin (*T*. *taczanowskii*), and the perch (*Lateolabrax japonicus*) have also been reported as the fish host (Chai, 2007, Chai et al., 2009a, Yu and Chai, 2013, Chai, 2015). The natural definitive hosts are humans (Chai et al., 2009a), dogs (Komiya, 1965, Cho et al., 1981), rats (Seo et al., 1981a), cats (Huh et al., 1993), foxes (Miyamoto, 1985), boars (Komiya, 1965), and kites (bird) (Miyamoto, 1985). Mice, rats, cats, dogs, gerbils, hamsters, and ducks are experimental definitive hosts (Guk et al., 2005, Chai et al., 2009a, Li et al., 2010, Li et al., 2013). Guk et al. (2005) studied on the growth and development of *M*. *yokogawai* in different strains of mice (BALB/c, ddY, C57BL/6J, C3H/HeN, and A/J) and found that ddY mice were the most highly suitable strain.

The principal mode of human infection is consumption of raw or improperly cooked freshwater fish, notably the sweetfish (*P*. *altivelis*) and the big-scaled redfin (*T*. *hakonensis*) (under the name *T*. *taczanowskii*) (Yu and Chai, 2013, Chai, 2015). Pickled, salted, or fermented fish, as well as cooking knife and chopping board contaminated with the metacercariae may also cause human infections (Chai, 2015). Major endemic areas are scattered in Far Eastern countries, including the Republic of Korea (= Korea), Japan, China, and Russia (Chai, 2015). In Korea, numerous endemic foci have been found, and almost all small streams to large rivers in eastern and southern coastal areas were confirmed to be endemic areas (Chai and Lee, 2002, Chai, 2007, Chai, 2015, Chai, 2017, Chai et al., 2009a). High endemic areas are the Seomjin, Tamjin, Boseong Rivers, Geoje Island, and Osip Stream in Samcheok-shi (Gangwon-do) which revealed 20–70% egg positive rates of the riparian residents (Chai and Lee, 2002, Chai et al., 1977, Chai et al., 2000, Chai et al., 2015a). Of considerable interest in Korea is that the eggs of *M*. *yokogawai* were detected from mummies of the 17th century, Joseon Dynasty (Seo et al., 2008, Shin et al., 2009). It is thus presumed that the life cycle of *M*. *yokogawai* has been actively maintained at least more than 400 years in Korea (Seo et al., 2008).

In Japan, it was originally thought that *M*. *yokogawai* infection is distributed nationwide with the exception of Hokkaido (Ito, 1964a). However, the presence of its life cycle in Hokkaido was also recognized (Miyamoto, 1985). Until the 1960s, the prevalence in humans ranged 0.5–35.1% depending on the locality surveyed (Ito, 1964a). Kagei and Kihata (1970) surveyed 26 areas of Japan and reported *M*. *yokogawai* egg positive rates as 0–73.9%; three areas of Shimane Prefecture showed the highest prevalence (73.9%, 71.9%, and 57.1%, respectively) followed by areas of Hiroshima (38.9%), Kochi (33.2%), Kagoshima (28.0%), and Saga Prefecture (27.0%). The last available literature on the prevalence (Ito et al., 1991) reported 13.2% egg positive rate among 4524 riparian people examined in Hamamatsu Lake, Shizuoka Prefecture during 1982–1988. It is presumed that the prevalence of *M*. *yokogawai* in Japan has been decreasing until now. In China, 8 human infections were confirmed by genetic analysis of fecal eggs in Guangxi Province (Jeon et al., 2012). It was stated that human infections exist in Guangdong, Anhui, Hubei, and Zhejiang Province (Yu and Mott, 1994). In Taiwan, the metacercariae of *M*. *yokogawai* were first found from the sweetfish in 1912 (Yokogawa, 1912, Ito, 1964a), and after then, human infections were occasionally reported (Chen, 1991). However, little surveys have been undertaken on the prevalence among humans (Li et al., 2013).

In Russia, the Amur and Ussuri valleys of the Khabarovsk territory have been known to be endemic areas of *M*. *yokogawai* (Yu and Mott, 1994). In this territory, the prevalence among the total population was 1–2% but the prevalence among the ethnic minority was 20–70% (Yu and Mott, 1994). Also in the north of Sakhalin Island, the infection rate was 10% among ethnic minorities and 1.5% among Russians (Yu and Mott, 1994). Sporadic cases were also reported in the Amur district and the Primorye territory (Yu and Mott, 1994, Chai, 2015). In the Khabarovsk territory, the population at risk was once estimated at 859,000, which is 14.7% of the total population in this territory (Yu and Mott, 1994). In India, the presence of human infection was suspected because two heterophyid egg positive cases (designated as *M*. *yokogawai* infection) were detected (Mahanta et al., 1995, Uppal and Wadhwa, 2005). In Europe, the existence of *M*. *yokogawai* infection (in some references described as *Metagonimus* sp.) have been reported in fish hosts and wild animals of Ukraine (Davydov et al., 2011), Serbia (Djikanovic et al., 2011), Bulgaria (Nachev and Sures, 2009, Ondračkova et al., 2012), and Czech Republic (Francová et al., 2011). However, human infections have not yet been confirmed.

#### 2.1.2 *Metagonimus takahashii* (Takahashi, 1929) Suzuki, 1930

This species was originally found from the small intestine of mice and dogs fed the metacercariae encysted in freshwater fish in Japan (Takahashi, 1929a). A year later, it was proposed as a new species *M*. *takahashii* admitting that the large egg size is enough to be a specific character (Suzuki, 1930). Its taxonomic validity had long been debated by Japanese parasitologists. However, Saito, 1972, Saito, 1973 strongly supported the validity of *M*. *takahashii* based not only on its remarkably larger egg size but also on differential morphologies of larval and adult stages and also by the different host specificities at experimental infection with the cercariae. Thereafter, the name *M*. *takahashii* has been settled. *M*. *takahashii* differs morphologically from *M*. *yokogawai* and *M*. *miyatai* in the position of the two testes, distribution of vitelline follicles, and size of their eggs (Saito, 1984, Chai et al., 1993a, Chai, 2015) (Fig. 2). It also differs from *M*. *katsuradai*, *M*. *otsurui*, and *M*. *hakubaensis* in that the latter three species have a smaller ventral sucker than the oral sucker (Saito, 1984, Shimazu, 1999, Chai, 2015). *M*. *minutus*, having a larger ventral sucker than the oral sucker, differs from *M*. *takahashii* and *M*. *yokogawai* in having a smaller body and eggs, larger seminal vesicle, and different fish host (Katsuta, 1932a, Chai et al., 1993a).

The snail hosts are *Semisulcospira* spp. (including *S*. *libertina* and *S*. *coreana*) (Saito, 1972, Cho et al., 1984). The fish hosts include the crussian carp (*Carassius auratus*), carp (*Cyprinus carpio*), big-scaled redfin (*T*. *hakonensis*), and perch (*L*. *japonicus*) (Takahashi, 1929a, Saito, 1984, Sohn, 2009, Chai, 2015). The natural definitive hosts are mice, rats, dogs, cats, pelicans, kites, and other avian species (Takahashi, 1929a, Yamaguti, 1958, Ahn, 1993). The experimental definitive hosts include mice, rats and dogs, cats, hamsters, and rabbits (Takahashi, 1929a, Saito, 1972, Chai et al., 1991, Rim et al., 1996, Guk et al., 2005, Chai, 2017). The growth and development of *M*. *takahashii* were studied in different strains of mice (BALB/c, ddY, C57BL/6J, C3H/HeN, and A/J), that were generally not a good experimental host (Guk et al., 2005).

This species is known to distribute only in Korea and Japan. However, it is possibly distributed in other countries. In Korea, *M*. *takahashii* was first found in experimental rabbits fed the metacercariae from carps (Chun, 1960a). The presence of human infections (mixed with *Metagonimus* sp., presumably *M*. *miyatai*) was first demonstrated by adult worm recovery from riparian people along the Hongcheon River, Gangwon-do (Ahn and Ryang, 1988). An endemic area was subsequently discovered along the upper reaches of the Namhan River, mixed-infected with *M*. *miyatai*, with an egg positive rate of 9.7% for both species (Chai et al., 1993a). A recent survey performed along the Boseong River, Jeollanam-do (*M*. *yokogawai* endemic area) detected 3293 specimens of *M*. *takahashii* from 11 riparian residents out of a total of 70,223 intestinal fluke specimens (mostly *M*. *yokogawai*) (Chai et al., 2015a). In Japan, articles regarding its existence have been published (Ito, 1964a, Saito, 1972, Saito, 1973). However, because of taxonomic debates and confusion between *M*. *takahashii* and *M*. *yokogawai*, its precise epidemiologic status, including the prevalence and geographical distribution of human infections, has not been clearly defined (Chai, 2015). An earlier study in Okayama City reported infection of *M*. *takahashii* in 43 (0.64%) of 6680 residents examined, whereas infection of *M*. *yokogawai* was found in 54 (0.81%) residents (Takahashi, 1929a). Later, in Fuchu City, Hiroshima Prefecture, 11 (4.8%) of 231 residents examined were infected with *M*. *takahashii*, whereas 81 (35.1) were infected with *M*. *yokogawai* (Asada et al., 1957).

#### 2.1.3 *Metagonimus miyatai* Saito, Chai, Kim, Lee, and Rim, 1997

Saito et al. (1997) described *M*. *miyatai* as a new species based on adult flukes collected from dogs and hamsters experimentally fed the metacercariae from the sweetfish (*P*. *altivelis*), dace (*T*. *hakonensis* and *T*. *taczanowskii*), Amur fat-minnow (*Phoxinus steindachneri*) (under the name *Morocco steindachneri*), pale chub (*Zacco platypus*), and dark chub (*Zacco temminckii*) in Korea and Japan. This species was actually first found by Katsurada (1912) (shown only by a figure drawing) together with *M*. *yokogawai* in Taiwan, but at that time it was regarded as a paratype specimen of *M*. *yokogawai* (Saito et al., 1997). Human infections have been reported from Korea and Japan (Saito et al., 1997, Chai et al., 1993a, Chai et al., 2015a). *M*. *miyatai* is morphologically characterized by two markedly separated testes from each other, with the posterior one located very close to the posterior body wall, vitelline follicles never distributing beyond the posterior testis, and the egg size which is intermediate between those of *M*. *yokogawai* and *M*. *takahashii* (Chai et al., 1993a, Saito et al., 1997, Yu and Chai, 2013, Chai, 2015) (Fig. 2). It differs from *M*. *minutus* in its larger body and egg size (Katsuta, 1932a, Chai et al., 1993a). *M*. *miyatai* is also genetically distinct from *M*. *yokogawai* and *M*. *takahashii* (Yu et al., 1997a, Yu et al., 1997b, Lee et al., 1999, Yang et al., 2000).

The snail host includes *S*. *libertina*, *S*. *dolorosa*, *S*. *globus*, and *Koreanomelania nodifila* (Kim, 1980, Kim et al., 1987, Shimazu, 2002). The metacercariae have been detected in freshwater fish (including *Z*. *platypus*, *Z*. *temminckii*, *P*. *altivelis*, *T*. *hakonensis*, *T*. *taczanowskii*, *Opsariichthys bidens*, and *Phoxinus steindachneri*) (Saito et al., 1997, Shimazu, 2002, Sohn, 2009). Natural definitive hosts include the dog, red fox, raccoon dog, and black-eared kite (Saito et al., 1997, Chai, 2015). Experimental definitive hosts are the mouse, rat, hamster, and dog (Kim, 1980, Kim et al., 1987, Chai et al., 1991, Ahn and Ryang, 1995, Saito et al., 1997, Shimazu, 2002, Chai, 2017). The worm growth and development was studied in different strains of mice (BALB/c, ddY, C57BL/6J, C3H/HeN, and A/J); mice were generally not a susceptible animal host (Guk et al., 2005).

The major source of human infection is raw or improperly cooked freshwater fish, in particular, the pale chub (*Z*. *platypus*) in Korea and Japan (Chai, 2015). In Korea, the presence of human infections (under the name *Metagonimus* sp.) was first demonstrated by Kim (1980) in Geum River by detecting eggs in the feces and recovery of adult flukes from several cases. Kim et al. (1987) performed another survey around the Lake Daecheong and its upper reaches (designated the worms as *Metagonimus* Miyata type of Saito, 1984) and reported *Z*. *platypus* and *Opsariichthys bidens* as the most heavily infected fish species. Adult specimens were recovered from 32 people living along the Namhan River in Eumseong-gun (9.7% in egg positive rate) and Yeongwol-gun (48.1% in egg positive rate) (Chai et al., 1993a). A most recent survey performed along the Boseong River, Jeollanam-do (*M*. *yokogawai* endemic area) detected 343 specimens of *M*. *miyatai* from 11 riparian residents out of a total of 70,223 intestinal fluke specimens (mostly *M*. *yokogawai*) (Chai et al., 2015a). In Japan, epidemiological studies, particularly on human infections, are scarce. With regard to animal definitive hosts, Saito et al. (1997) listed dogs, foxes, raccoon dogs, and black-eared kites in Shimane, Kochi, and Yamagata Prefectures. Metacercariae were detected in a fish species (*P*. *steindachneri*) in Hiroi River basin, Nagano Prefecture (Shimazu, 2002). Small rivers of Shizuoka Prefecture were also found to have *M*. *miyatai* metacercariae-infected fish (Kino et al., 2006).

#### 2.1.4 *Metagonimus minutus* Katsuta, 1932

The original description of this species is based on adult flukes recovered from cats and mice experimentally fed the metacercariae in the brackish water mullet in Taiwan (Katsuta, 1932a). It is smaller than *M*. *yokogawai*, *M*. *takahashii*, and *M*. *miyatai* in its egg and adult worm size (Katsuta, 1932a, Ito, 1964a, Saito et al., 1997). Its body size is slightly larger than *M*. *katsuradai* but its egg size is smaller than *M*. *katsuradai* (Katsuta, 1932a, Izumi, 1935). Its oral sucker is smaller than the ventral sucker, whereas, in *M*. *katsuradai*, the oral sucker is bigger than the ventral sucker (Katsuta, 1932a, Izumi, 1935, Chai, 2015). This species has been listed as a human-infecting intestinal fluke species in Taiwan but the literature background is not available (Yu and Mott, 1994).

The molluscan host is unknown. The metacercariae are found in the scales, gills, and fins of the mullets (*Mugil cephalus*) (Katsuta, 1932a). Natural definitive hosts have not been discovered. Cats and mice were used as experimental definitive hosts (Katsuta, 1932a). Eating raw or improperly cooked mullets in endemic areas is a risk factor. Distribution of this species in other countries is unknown.

#### 2.1.5 *Metagonimus katsuradai* Izumi, 1935

The original description of this species is based on adult flukes recovered from rats, mice, rabbits, dogs, and cats experimentally infected with the metacercariae obtained from freshwater fish (including *Pseudorasbora parva*, *Z*. *platypus*, and *Tanakia lanceolata*) in Japan (Izumi, 1935). The possibility of human infection was experimentally proven through infection to author himself and family (Izumi, 1935). The body of *M*. *katsuradai* is slightly smaller than *M*. *minutus* but its eggs are larger than *M*. *katsuradai* (Katsuta, 1932a, Izumi, 1935). It differs from *M*. *yokogawai*, *M*. *takahashii*, *M*. *miyatai*, and *M*. *minutus* in having a smaller ventral sucker than the oral sucker (Yu and Chai, 2013, Chai, 2015). It also differs from *M*. *otsurui* in the position of the seminal receptacle (Saito and Shimizu, 1968). It differs from *M*. *hakubaensis* in its long ceca that enter the post-testicular region (Shimazu, 1999).

The molluscan host is *S*. *libertina* in Japan (Kurokawa, 1939) and *Juga tegulata* in Russia (Besprozvannykh et al., 1987). The second host is freshwater fish (including *Tanakia lanceolata*, *T*. *oryzae*, *T*. *limbata*, *Acheilognathus rhombeus*, *T*. *moriokae*, *P*. *parva*, *Z*. *platypus*, and *Gnathopogon elongatus*) (Izumi, 1935, Ito, 1964a, Shimazu, 2003). Dogs are the only found natural definitive host (Yoshikawa et al., 1940). Experimental definitive hosts include humans, mice, white mice, rats, rabbits, puppies, kittens, ducks, and golden hamsters (Izumi, 1935, Kurokawa, 1939, Shimazu, 2003, Chai, 2017). Consumption of raw or improperly cooked freshwater fish (*Z*. *platypus* and others) is a risk factor (Ito, 1964a).

In Japan, its presence has been reported in several localities (Kurokawa, 1939, Ito, 1964a, Urabe, 2003). However, natural human cases have never been documented. In Russia, the existence of its life cycle was reported by Besprozvannykh et al. (1987) in the southern Primorye region through discovery of cercariae in *Juga* snails and metacercariae in 6 species of freshwater fish. Without firm evidence, it is listed among the human-infecting trematodes in the Primorye Territory (Emolenko et al., 2015).

### 2.2 Heterophyes

Flukes of *Heterophyes* are characterized by the presence of a genital sucker and armed gonotyl; they differ from *Metagonimus* in having a large median located ventral sucker and from *Haplorchis* in having only one testis (Chai, 2007). *Heterophyes* flukes were first discovered by Bilharz in 1851 at an autopsy of an Egyptian and named as *Distomum heterophyes* by von Siebold in 1852 (Chai, 2007, Chai, 2014b). The genus *Heterophyes* was raised by Cobbold in 1866 with *Heterophyes aegyptiaca* as the type; later this was synonymized with *H*. *heterophyes* (Witenberg, 1929). A total of 18 species or subspecies had been described in the genus *Heterophyes* (Chai, 2014b). However, three species were moved to another genus *Alloheterophyes* by Pearson (1999) and nine species were synonymized with *H*. *heterophyes* or other pre-existing species (Chai, 2014b). Now, the number of valid species of *Heterophyes* is only six (Pearson, 1999, Chai, 2014b), namely, *H*. *heterophyes* (by von Siebold in 1852), *H*. *nocens* (Onji and Nishio, 1916), *H*. *dispar* (Looss, 1902), *H*. *aequalis* (Looss, 1902), *H*. *indica* (Rao and Ayyar, 1931), and *H*. *pleomorphis* (Bwangamoi and Ojok, 1977) (Table 2). Four species, *H*. *heterophyes*, *H*. *nocens*, *H*. *dispar*, and *H*. *aequalis*, are known to infect humans (Ashford and Crewe, 2003, Chai, 2014b). Caution is needed to refer to some old literature which dealt with *H*. *nocens* as a synonym of *H*. *heterophyes*.

**Table 2:** Species of *Heterophyes* reported in the literature.

Species	Human infection	Country/region	First reporter
*Heterophyes heterophyes*	Yes	Egypt, Sudan, Palestine, Turkey, India, Middle East, Japan^a^, Korea^a^	von Siebold in 1852 (Ransom, 1920)
*Heterophyes nocens*	Yes	Korea, Japan	Onji and Nishio (1916)
*Heterophyes dispar*	Yes	Egypt, Middle East, Korea^b^	Looss (1902)
*Heterophyes aequalis*	Yes	Egypt, Middle East	Looss (1902)
*Heterophyes indica*	No	India	Rao and Ayyar (1931)
*Heterophyes pleomorphis*	No	Uganda	Bwangamoi and Ojok (1977)
Twelve other species (subspecies)^c^	No		

a Imported human cases were reported in Japan (Kagei et al., 1980) and Korea (Eom et al., 1985a, Chai et al., 1986b).

b Imported human cases were reported in Korea (Eom et al., 1985a, Eom et al., 1985b, Chai et al., 1986a, Chai et al., 1986b).

c Six of them, including *H*. *aegyptiaca*, *H*. *fraternus*, *H*. *persicus*, *H*. *heterophyes sentus*, *H*. *inops*, and *H*. *palidus*, were synonymized with *H*. *heterophyes* (Witenberg, 1929). *H*. *dispar limatus* was synonymized with *H*. *dispar* (Witenberg, 1929). The validity of *H*. *elliptica* has been questioned (Waikagul and Pearson, 1989). *H*. *katsuradai* was synonymized with *H*. *nocens* (Witenberg, 1929). *H*. *superspinata* (syn. *H*. *bitorquatus*) and *H*. *chini* have been transferred to another genus *Alloheterophyes* (Pearson, 1999).

#### 2.2.1 *Heterophyes heterophyes* (v. Siebold, 1852) Stiles and Hassal, 1900

**Fig. 3:**
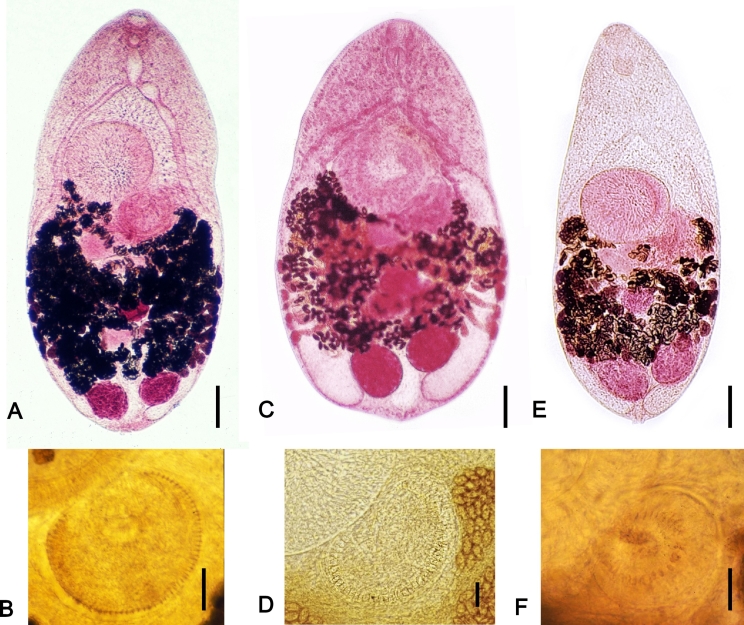
Morphology of *Heterophyes* spp. infecting humans. (A, B) *Heterophyes heterophyes*, (C, D) *Heterophyes nocens*, and (E, F) *Heterophyes dispar*. Note that *H*. *heterophyes* has a gonotyl armed with more than 70 chitinous spines (rodlets) around the genital sucker (B), whereas *H*. *nocens* and *H*. *dispar* have about 60 (D) and 30 rodlets (F), respectively. Scale bars; (A) = 100 μm, (B) = 100 μm, (C) = 100 μm, (D) = 10 μm, (E) = 10 μm, (F) = 10 μm. The photo (A) has been reproduced, with permission, from the black and white photo of the same specimen reported by Chai et al. (1986b) in Korean J. Parasitol. 24, 82–88.

This species was first discovered by Bilharz in 1851 at autopsy of an Egyptian in Cairo (Chai, 2007, Chai, 2014b). It is now known to cause human infections along the Nile Delta of Egypt and Sudan, the Middle East, southeastern Europe, and India (Yu and Mott, 1994, Mahanta et al., 1995, Pica et al., 2003, Chai et al., 2005a). The adult flukes are minute, ovoid to elliptical, elongate, or pyriform in shape (Witenberg, 1929). Their unique morphologies include the presence of two side-by-side testes near the posterior extremity of the body, a large ventral sucker which is located median, and a large submedian genital sucker armed with 70–85 chitinous rodlets on the gonotyl (Chai, 2007, Chai, 2014b) (Fig. 3). Adults of *H*. *heterophyes* differ from those of *H*. *nocens* mainly in the number of rodlets on the gonotyl; 50–62 in *H*. *nocens* and 70–85 in *H*. *heterophyes* (Witenberg, 1929, Chai et al., 1994b, Chai et al., 2009a). Adults of *H*. *dispar* are slightly smaller in body size and have smaller sizes of the genital sucker and smaller numbers of rodlets on the gonotyl (22–35) compared with those of *H*. *heterophyes* and *H*. *nocens* (Witenberg, 1929, Chai et al., 1986a, Chai et al., 2009a).

The eggs contain a fully developed miracidium and hatch after ingestion by an appropriate freshwater snail (for example, *Pirenella conica*) (Taraschewski, 1984). The cercariae enter between the scales of freshwater or brackish water fish, including mullets (*Mugil cephalus*, *Mugil capito*, *Mugil auratus*, *Mugil saliens*, and *Mugil chelo*), *Tilapia* fish (*Tilapia nilotica* and *Tilapia zilli*), and gobies/other fish (*Aphanius fasciatus*, *Barbus canis*, *Sciaena aquilla*, *Solea vulgaris*, and *Acanthogobius* sp.), and encyst chiefly in the muscle of these fish host (Paperna and Overstreet, 1981, Velasquez, 1982). Various species of mammals and birds, including dogs, cats, wolves, bats, rats, foxes, sea gulls, and pelicans, act as the natural definitive hosts and take the role of reservoir hosts (Yamaguti, 1958, Chai, 2014b). Rats, dogs, cats, foxes, badgers, pigs, macaques, and gulls can be experimental definitive hosts (Chai, 2014b).

The principal mode of human infections is consuming raw or improperly cooked fish, notably mullets and gobies (Chai, 2014b). Human and/or animal infections have been reported in Egypt, Sudan, Greece, Turkey, Palestine, Italy, Tunisia, India, and the Middle East, including Saudi Arabia, Iran, Iraq, United Arab Emirates, Kuwait, and Yemen (Yu and Mott, 1994, Chai, 2014b, Tandon et al., 2015). About 30 million people are estimated to be infected with this fluke (Mehlhorn, 2015). In Egypt, human infections are commonly found in the northern part of the Nile Delta, particularly around Lakes Manzala, Burullus, and Edku, where fishermen and domestic animals frequently consume fish (Yu and Mott, 1994, Youssef and Uga, 2014). In an earlier report, an extremely high prevalence (88%) was found among schoolchildren at Mataria on Lake Manzala (Khalil, 1933). In Dakahlia Governorate, the disease was common in both urban and rural localities owing to the habit of consuming salted or insufficiently baked fish (Sheir and Aboul-Enein, 1970). After 1960s–1980s, the prevalence began to decline down to 2.5–10.0% in these areas (Rifaat et al., 1980, Youssef et al., 1987a). However, near the Lake Edku, the prevalence among 2219 individuals was 33.8% demonstrating still considerably high endemicity in northern parts of Nile Delta (Abou-Basha et al., 2000). Interestingly, some Japanese immigrants living in Egypt were found positive for heterophyid eggs (including *H*. *heterophyes*); the prevalence was 11% in 2005, 11% in 2006, 15% in 2007, and 14% in 2008 (Okuzawa et al., 2010).

In Sudan, few epidemiological surveys have been undertaken, although some humans (Koreans) who lived in Sudan and returned home were found infected with *H*. *heterophyes* and *H*. *dispar* (Eom et al., 1985a). In Iran, the prevalence of heterophyid (including *H*. *heterophyes*) infections in humans and animals was documented by Massoud et al. (1981); in 13 villages of Khuzestan, the prevalence ranged 2–24%. In Saudi Arabia, Kuwait, Dubai, and United Arab Emirates, no reports are available on human infections. However, the existence of *H*. *heterophyes* has been documented in fish intermediate hosts or animal reservoir hosts (Abdul-Salam and Baker, 1990, Khalil et al., 2014, El-Azazy et al., 2015). Sporadic *H*. *heterophyes* infection has been reported from Greece, Turkey, Italy, Spain, Tunisia, Yemen, and Sri Lanka (Taraschewski and Nicolaidou, 1987, Yu and Mott, 1994, Martínez-Alonso et al., 1999). Imported human cases were reported in France (Rousset and Pasticier, 1972), Korea (Eom et al., 1985a, Chai et al., 1986a), and Japan (Kagei et al., 1980); the patients returned from Egypt, Saudi Arabia, or Sudan. In USA, a woman patient was found to be infected with *H*. *heterophyes* (the species is uncertain because only eggs were detected in her stool) after eating ‘sushi’ in a local Japanese restaurant (Adams et al., 1986).

#### 2.2.2 *Heterophyes nocens* Onji and Nishio, 1916

The original description of this species is based on adult flukes recovered from experimental dogs and cats fed the metacercariae encysted in mullets *Mugil cephalus* in Japan (Onji and Nishio, 1916). Later, *Heterophyes katsuradai* was recovered from a man in Kobe, Japan and described as a new species (Ozaki and Asada, 1926). However, it was synonymized with *H*. *nocens* (Witenberg, 1929, Waikagul and Pearson, 1989). It is now known to occur in Korea, Japan, China, Taiwan (in China and Taiwan, the name was written as *H*. *heterophyes* probably by an error), and Thailand (Yokogawa et al., 1965, Seo et al., 1981b, Chai et al., 1984, Chai et al., 1985, Yu and Mott, 1994, Namchote et al., 2015). Its adult flukes are elongate, elliptical, or pyriform, and morphologically close to *H*. *heterophyes* (Chai et al., 1986b, Chai et al., 1994b). The only recognizable difference is the smaller number of rodlets on the gonotyl of the genital sucker in *H*. *nocens* (50–62) compared to that in *H*. *heterophyes* (70–85) (Taraschewski, 1984, Chai et al., 1986b, Chai et al., 1994b, Chai and Lee, 2002) (Fig. 3). *H*. *dispar* has a slightly smaller body, a smaller-sized genital sucker, and a smaller number (22–35) of rodlets on the gonotyl compared to *H*. *nocens* and *H*. *heterophyes* (Witenberg, 1929, Chai et al., 1986b). *H*. *aequalis* is also smaller in body size than *H*. *nocens* and *H*. *heterophyes*, has relatively short ceca, and has a smaller number (14–25) of rodlets on the gonotyl (Witenberg, 1929, Chai et al., 1986b).

The eggs hatch after ingestion by an appropriate brackish water snail, for example, *Cerithidea cingulata* (=* Tympanotonus microptera*) or *Cerithidea fluviatilis* (Ito, 1964b). The cercariae enter between the scales of brackish water fish, including mullets and gobies (*Mugil cephalus*, *Liza menada*, *Tridentiger obscurus*, *Glossogobius brunnaeus*, *Therapon oxyrhynchus*, and *Acanthogobius flavimanus*) (Ito, 1964a, Komiya, 1965, Sohn, 2009). Two species of brackish water gobies (*Boleophthalmus pectinirostris* and *Scartelaos* sp.) can also host *H*. *nocens* (Sohn et al., 2005). Cats, dogs, and rats are natural definitive hosts (Yoshikawa et al., 1940, Ito, 1964a, Seo et al., 1981a, Eom et al., 1985b, Sohn and Chai, 2005, Shin et al., 2015). Mice and rats can be used as experimental definitive hosts (Chai et al., 2009a, Chai, 2014b).

Raw or improperly cooked mullets and gobies are the major source of human infections (Chai, 2014b). Human infections have been reported in Korea, Japan, China, and Taiwan (Yu and Mott, 1994). In Korea, about 50,000 people are estimated to be infected with this fluke (Chai and Lee, 2002). In Korea, human infection was first identified in a man residing in a seashore village along the Western Sea (= Yellow Sea) (Seo et al., 1981b). Thereafter, cases were reported from western and southern coastal areas (Chai et al., 1984, Chai et al., 1985). Subsequently, numerous endemic areas with 10–40% prevalences were detected in southwestern coastal areas, i.e., Jeollanam-do and Gyeongsangnam-do Province (Chai et al., 1994b, Chai et al., 1997, Chai et al., 1998, Chai et al., 2004, Park et al., 2007, Guk et al., 2007). In addition, in an inland area of Boseong-gun (along the Boseong River) where *M*. *yokogawai* is highly endemic, a few adult specimens of *H*. *nocens* were also recovered from some riparian residents (Chai et al., 2015a).

In Japan, an earlier study reported 18.5% prevalence among inhabitants in Yamaguchi Prefecture (Onji, 1915). Later, in Chiba Prefecture, the heterophyid egg positive rate was 8.0% among residents in Goi-Machi village and 9.0% in Misaki-Machi village (Yokogawa et al., 1965). In Shizuoka Prefecture, the prevalence of *H*. *nocens* was 20.7% in Harai village of Izu peninsula but six years after a mass treatment, inhibition of raw fish eating, and health education, the prevalence decreased to 3.4% (Ito et al., 1967). Human *H*. *nocens* infection was also reported from Chugoku and Hiroshima Prefectures (Suzuki et al., 1982). Two lakeside villages of Mikkabi-cho, north end of Hamana Lake, Shizuoka Prefecture were also found to have 7.5% and 10.5% prevalences of *H*. *nocens* eggs (Kino et al., 2002). In China, human infections (under the name *H*. *heterophyes*) were reported in provinces of mainland China (Guangdong, Hubei, and Beijing) and Taiwan (Yu and Mott, 1994). However, the parasite may have been actually *H*. *nocens* (Chai, 2014b). The life cycle of *H*. *nocens* may be present in Thailand, since cercariae of *Heterophyes* sp. were recovered from brackish water snails (Namchote et al., 2015). A French professor who visited Japan and consumed raw fish and aquatic plants there was found to be infected with *H*. *nocens* (under the name *H*. *heterophyes*) (Lamy et al., 1976).

#### 2.2.3 *Heterophyes dispar* Looss, 1902

The original description is based on specimens discovered in the intestines of dogs and cats in Egypt (Looss, 1902). It was found in other mammals, including foxes and wolves, in the northern Africa and eastern Mediterranean, including Greece and Palestine (Witenberg, 1929, Taraschewski and Nicolaidou, 1987, Yu and Mott, 1994, Chai, 2014b). Human infections were first reported from 2 Korean men returning from Saudi Arabia (Chai et al., 1986a) and then also found in Kalasin Province, Thailand (Yu and Mott, 1994). There were taxonomic debates on *H*. *dispar* and *H*. *aequalis*, both of which were described by Looss (1902) in Egypt. However, *H*. *dispar* is distinct from *H*. *aequalis* in the larger number of rodlets (22–35) on the gonotyl compared with *H*. *aequalis* (14–25) and long ceca extending down to the posterior margin of two testes in *H*. *dispar*, whereas they are ending before the anterior margin of two testes in *H*. *aequalis* (Looss, 1902, Witenberg, 1929). Based on several differential characters, Taraschewski, 1985a, Taraschewski, 1985b, Taraschewski, 1987 acknowledged the validity of both species. Adults of *H*. *dispar* are elliptical or pyriform, and slightly smaller than those of *H*. *heterophyes* and *H*. *nocens* (Chai et al., 1986a, Chai, 2014b). A remarkable difference is the smaller number of rodlets on the gonotyl of the genital sucker in *H*. *dispar* (22–35) compared to that in *H*. *heterophyes* (70–85) and *H*. *nocens* (50–62) (Chai et al., 1986a, Chai et al., 1994b, Chai, 2014b) (Fig. 3).

Freshwater or brackish water snails (*P*. *conica*) serve as the first intermediate host, and various species of freshwater or brackish water fish (including *Mugil* spp., *Epinephelus enaeus*, *Tilapia* spp., *Lichia* spp., *Barbus canis*, *Solea vulgaris*, and *Sciaena aquilla*) are the second intermediate hosts (Witenberg, 1929, Paperna and Overstreet, 1981, Taraschewski, 1984). Natural definitive hosts include dogs, cats, wolves, jackals, foxes, and kites (Witenberg, 1929, Chai, 2014b). Pups, rabbits, rats, cats, and red foxes are experimental definitive hosts (Taraschewski, 1985b).

Human infections may occur when *Mugil* spp. fish are eaten raw or under inadequately cooked conditions (Chai, 2014b). However, human infections were unknown before 1985–1986 when some Korean men were found infected with this fluke together with *H*. *heterophyes* who returned from Sudan (Eom et al., 1985a) and Saudi Arabia (Chai et al., 1986a). Human infectins were also confirmed in Thailand (Yu and Mott, 1994).

#### 2.2.4 *Heterophyes aequalis* Looss, 1902

The original description is based on specimens discovered in the intestines of dogs and cats in Egypt (Looss, 1902). It infects various other mammals too (Witenberg, 1929, Taraschewski, 1985b). The presence of human infections was first mentioned by Kahlil in Egypt in 1991 (Ashford and Crewe, 2003), although the literature background is difficult to obtain. Morphologically *H*. *aequalis* is most similar to *H*. *dispar* but distinct from it in the smaller number of rodlets (14–25) on the gonotyl in comparison with *H*. *dispar* (22–35) and short ceca ending extending before the anterior margin of two testes in *H*. *aequalis*, whereas they reach down to the posterior margin of two testes in *H*. *dispar* (Looss, 1902, Witenberg, 1929).

Brackish water snails (*P*. *conica*) serve as the first intermediate host (Taraschewski, 1984). Freshwater or brackish water fish (including *M*. *cephalus*, *M*. *capito*, *M*. *auratus*, *E*. *enaeus*, *T*. *simonis*, *L*. *amia*, *L*. *glauca*, and *B*. *canis*) are the second intermediate hosts (Witenberg, 1929). Natural definitive hosts include cats, dogs, Persian wolves, ref. foxes, rats, pigs, pelicans, kites, and herons (Witenberg, 1929, Taraschewski, 1985b). Pups, rabbits, rats, cats, and red foxes are experimental definitive hosts (Taraschewski, 1985b). Human infections may occur when *Mugil* spp. fish are eaten raw or under inadequately cooked conditions (Chai, 2014b). Human infections seem to occur in Egypt (Ashford and Crewe, 2003).

### 2.3 Haplorchis

Flukes of *Haplorchis* are characterized by the presence of only one testis and a small armed ventral sucker lacking a gonotyl; they differ from *Metagonimus* and *Heterophyes* in having only one testis (Chai, 2007). They lack the expulsor-style distal part of the seminal vesicle which is present in genera *Procerovum* and *Stellantchasmus* (Pearson and Ow-Yang, 1982). The genus *Haplorchis* was erected with *Haplorchis pumilio* as the type and *Haplorchis cahirinus* as another species (Looss, 1899). However, Witenberg (1929) transferred *H*. *cahirinus* to another family because it is an intestinal parasite of fish in its adult stage, whereas *H*. *pumilio* is an intestinal parasite of birds and mammals. At present, 9 species can be recognized as valid species in the genus *Haplorchis*. They include *H*. *pumilio* (Looss, 1899), *H*. *taichui* (Nishigori, 1924a), *H*. *yokogawai* (Katsuta, 1932b), *H*. *vanissimus* (Africa, 1938), *H*. *parataichui* (Pearson, 1964), *H*. *sprenti* (Pearson, 1964), *H*. *wellsi* (Pearson, 1964), *H*. *parapumilio* (Pearson and Ow-Yang, 1982), and *H*. *paravanissimus* (Pearson and Ow-Yang, 1982) (Table 3). Among them, *H*. *taichui*, *H*. *pumilio*, *H*. *yokogawai*, and *H*. *vanissimus* are human-infecting species. Human infections with *Haplorchis* spp. are prevalent in Southeast Asia, including countries located in Indo-China Peninsula, Taiwan, the Philippines, and also probably in Egypt (Chai, 2007).

**Table 3:** Species of *Haplorchis* reported in the literature.

Species	Human infection	Country/region	First reporter
*Haplorchis taichui*	Yes	Thailand, Laos, Malaysia, Philippines, India, Bangladesh, Vietnam, China, Taiwan, Egypt, Palestine	Nishigori (1924a)
*Haplorchis pumilio*	Yes	Thailand, Laos, Egypt, Palestine, India, Bangladesh, China, Taiwan, Cambodia, Philippines, Malaysia, Vietnam, Korea^a^	Looss in 1886 (Looss, 1902)
*Haplorchis yokogawai*	Yes	Thailand, Laos, Malaysia, Philippines, India, Indonesia, China, Taiwan, Cambodia, Vietnam, Australia, Egypt	Katsuta (1932b)
*Haplorchis vanissimus*	Yes	Philippines, Australia	Africa (1938)
*Haplorchis sprenti*	No	Australia	Pearson (1964)
*Haplorchis wellsi*	No	Taiwan	Pearson (1964)
*Haplorchis parataichui*	No	Australia	Pearson (1964)
*Haplorchis parapumilio*	No	Indonesia	Pearson and Ow-Yang (1982)
*Haplorchis paravanissimus*	No	Australia	Pearson and Ow-Yang (1982)

a A natural human case co-infected with *H*. *pumilio*, *Gymnophalloides seoi*, and *Gynaecotyla squatarolae* has been reported in Korea (Chung et al., 2011). However, Korea is tentatively not regarded as an endemic area for *Haplorchis* spp.

#### 2.3.1 *Haplorchis taichui* (Nishigori, 1924) Chen, 1936

**Fig. 4:**
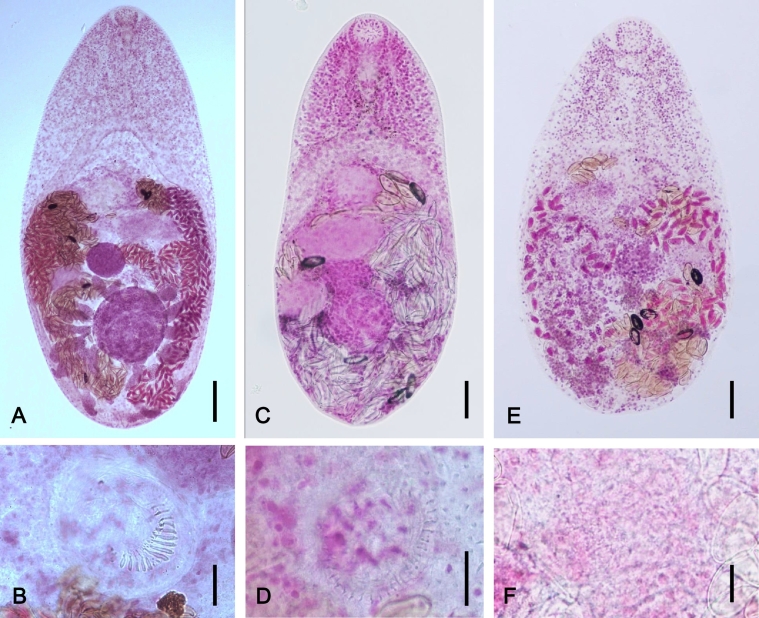
Morphology of *Haplorchis* spp. infecting humans. (A, B) *Haplorchis taichui*, (C, D) *Haplorchis pumilio*, and (E, F) *Haplorchis yokogawai*. Note that *H*. *taichui* has a gonotyl armed with 12–16 long and crescentic spines (B), whereas *H*. *pumilio* and *H*. *yokogawai* have about 32–40 minute sclerites (D) and numerous (uncountable) tiny spines (F), respectively. Scale bars; (A) = 100 μm, (B) = 100 μm, (C) = 100 μm, (D) = 10 μm, (E) = 10 μm, (F) = 10 μm.

The original description of this species was based on specimens recovered from birds and mammals caught in the middle part of Taiwan (Nishigori, 1924a). Its characteristic morphology (Fig. 4) includes a minute and oval body with flattened dorsal and ventral sides (Faust and Nishigori, 1926). The most specific morphological feature for differentiation from other *Haplorchis* species is the size, shape, and number of spines on the ventral sucker (Chen, 1936, Pearson and Ow-Yang, 1982). *H*. *taichui* has a semi-lunar group of 12–16 long, crescentic, and hollow spines (Fig. 4) and a sinistral patch of very minute solid spines (Pearson and Ow-Yang, 1982). Other species can be differentiated by the presence of minute sclerites on the ventral sucker (*H*. *pumilio*, *H*. *parapumilio*, *H*. *vanissimus*, and *H*. *paravanissimus*), remarkably large ventral sucker (*H*. *wellsi*), or a ventral sucker smaller than the oral sucker (*H*. *yokogawai* and *H*. *sprenti*) (Pearson and Ow-Yang, 1982).

The snail intermediate host is freshwater snails; *Melania obliquegranosa* in Taiwan (experimental) (Faust and Nishigori, 1926), *Melanoides tuberculata* and *Melania juncea* in the Philippines (Velasquez, 1973a), and *Tarebia granifera* in Hawaii (Martin, 1958). The cercariae enter between the scales of various species of freshwater fish (including *Barbodes gonionotus*, *Cirrhinus molitorella*, *Cyclocheilichthys* spp., *Hampala* spp., *Labiobarbus leptocheila*, *Mystacoleucus marginatus*, *Onychostoma elongatum*, *Puntius* spp., and *Rhodeus ocellatus*) (Velasquez, 1973b, Velasquez, 1982, Scholz et al., 1990, Rim et al., 2008). Birds and mammals, including dogs, cats, and humans, have been reported as the natural definitive hosts (Yamaguti, 1958). Experimental definitive hosts include mice, rats, hamsters, guinea pgs, rabbits, cats, and dogs (Faust and Nishigori, 1926, Sukontason et al., 2001). A human was also successfully infected with this fluke after swallowing the fish flesh (Faust and Nishigori, 1926).

In Thailand and Laos, fermentation is a traditional procedure for preservation of freshwater fish, and consumption of semi-fermented fish shortly after preparation increases the risk of human infections with *Haplorchis* spp. (Sukontason et al., 1998). Local fish dishes, notably ‘koi-pla’, ‘pla-som’, ‘pla-ra’, and ‘lab-pla’ are the major source of human infections (Sukontason et al., 1998). In the Philippines, a local fish dish called ‘kinilaw’ (freshwater fish seasoned only with salt and vinegar) is popularly consumed (Belizario et al., 2004). Other types of local fish dish in the Philippines include ‘sabaw’ (boiling fish for several minutes) and ‘sugba’ (grilling over charcoal) (Belizario et al., 2004). In Vietnam, raw or pickeld fish, for example, slices of silver carp, sold in Vietnamese restaurants are popularly consumed (Dung et al., 2007). *H*. *taichui* is known to distribute in Thailand, Laos, Cambodia, Vietnam, South China, Taiwan, the Philippines, Hawaii, Egypt, Palestine, Bangladesh, India, and Malaysia (Velasquez, 1982, Yu and Mott, 1994, Chai et al., 2005a, Chai et al., 2009a, Chai et al., 2014a).

In Thailand, northern parts, including Chiang Rai, Chiang Mai, Mae Hong Son, and Lamphun Provinces, were found to be endemic (Radomyos et al., 1998). In Phrae Province, the prevalence of *H*. *taichui* worms among 87 worm-recovery cases was 64.4% (Pungpak et al., 1998). In Nan Province, 37 of 50 praziquantel-treated patients were positive for *H*. *taichui* worms in their diarrheic stools, whereas in Lampang Province, 69 of 100 patients revealed *H*. *taichui* worms (Wijit et al., 2013). In northeastern parts, such as Khon Kaen Province, heterophyid trematodes are common among village people (Srisawangwong et al., 1997). In Laos, human *H*. *taichui* infection was first reported from 5 Laotians (Giboda et al., 1991). Thereafter, in fecal examinations of 29,846 Loatian people from 17 provinces and Vientine Municipality, the overall positive rate of small trematode eggs (*O*. *viverrini*, *H*. *taichui*, or other minute intestinal flukes) was 10.9% (3263 peole) (Rim et al., 2003). Chai et al. (2005b) performed worm recovery of *O*. *viverrini*, *H*. *taichui*, or other minute intestinal flukes from small trematode egg positive cases in two areas; Vientiane Municipality was highly prevalent with *O*. *viverrini* worms with a small number of intestinal flukes, including *H*. *taichui*, whereas Saravane Province was severely infected with *H*. *taichui* worms with a small number of *O*. *viverrini* specimens. Hyperendemicity of *H*. *taichui*, with high prevalence and heavy worm burden, was again documented in Saravane Province (Chai et al., 2013a). In Savannakhet Province, the prevalence of small trematode eggs among the riparian people was 67.1% (658/981), and the worms recovered after chemotherapy and purging of 29 egg-positive people consisted of similar numbers of *O*. *viverrini* (3347 worms) and *H*. *taichui* (2977 worms) (Chai et al., 2007). In Khammouane Province, the prevalence of small trematode eggs among the riparian people was higher than that in Savannakhet Province, 81.1% (1007/1242) (Chai et al., 2009b). An interesting finding in Phongsali Province was that the high prevalence (18.4%) of small trematode eggs notified (Rim et al., 2003) was confirmed to be exclusively due to *H*. *taichui* and *H*. *yokogawai* infections but not to *O*. *viverrini* infection (Chai et al., 2010). Also in Champasak and Luang Prabang Provinces, the infection was mostly due to intestinal flukes, in particular, *H*. *taichui*, *H*. *yokogawai*, and *H*. *pumilio* (Chai et al., 2013a, Chai et al., 2013b, Chai et al., 2013c, Sohn et al., 2014).

In Vietnam, a high prevalence (64.9%; 399/615) of minute intestinal trematodes, including *H*. *taichui*, *H*. *pumilio* (dominant species), and *H*. *yokogawai*, has been confirmed in human infections in two communes of Nam Dinh Province, a northern part of Vietnam (Dung et al., 2007). Later, in another commune in Nam Dinh Province, 22.7% (92/405) of household members were positive for small trematode eggs (De and Le, 2011). In Southern China, the presence of human *H*. *taichui* infection was first documented from Guangxi Province in 2004 (T. Li et al., 2010). Later, in several different areas of Guangxi Province, 28.0–70.6% prevalence was obtained by fecal examination for small trematode eggs; PCR analysis of the feces revealed that 29 of 46 egg positive cases were due to *H*. *taichui* (Jeon et al., 2012). In Taiwan, where *H*. *taichui* was originally discovered (Nishigori, 1924a, Faust and Nishigori, 1926), little study has been available on human infections with this fluke. In the Philippines, the Lake Lanao, Marawi City, Mindanao Island and U.P. rice paddies, Diliman, Quezon City, and Luzon Island have been listed as endemic areas of *H*. *taichui* (Velasquez, 1973b). In southern Mindanao, 36.0% (87/242) egg positive rate was reported from residents; *H*. *taichui* adult flukes were recovered from some of these people (Belizario et al., 2004). In Egypt and Kuwait, little has been reported regarding human infections; however, the presence of *H*. *taichui* was documented from animals (Kuntz and Chandler, 1956, El-Azazy et al., 2015).

#### 2.3.2 *Haplorchis pumilio* (Looss, 1896) Looss, 1899

The original description of this species is based on adult specimens recovered from the small intestine of birds and mammals in Egypt (Looss, 1899). Its specific morphological feature (Fig. 4) for differentiation from other *Haplorchis* species is the size, shape, and number of spines on the ventral sucker (Chen, 1936, Pearson and Ow-Yang, 1982). *H*. *pumilio* has a circle of 32–40 I- or Λ-shaped sclerites (Fig. 4), 2.5–5.9 μm long, interrupted dorsally between latero-dorsal lobes of the ventral sucker, and a few to 9 small simple spines of various lengths on latero-dorsal and mid-dorsal lobes, respectively (Pearson, 1964, Pearson and Ow-Yang, 1982). *H*. *taichui* is differed from *H*. *pumilio* by the presence of a semi-lunar group of 12–16 long, crescentic, and hollow spines and a sinistral patch of very minute solid spines. Other species can be differed from *H*. *pumilio* by the presence of 15–21 hollow spines (*H*. *parataichui*), ventral sucker with four lobes and armed with spines (*H*. *vanissimus*), ventral sucker without lobes and spines interrupted mid-ventrally (*H*. *paravanissimus*), remarkably large ventral sucker (*H*. *wellsi*), or a ventral sucker smaller than the oral sucker (*H*. *yokogawai* and *H*. *sprenti*) (Pearson and Ow-Yang, 1982).

The snail host is *Melania reiniana* var. *hitachiens* (experimental) in Taiwan (Faust and Nishigori, 1926, Velasquez, 1982) and *Melanoides* (=* Thiara*) *tuberculata* (natural) in Taiwan (Lo and Lee, 1996, Wang et al., 2002), India (Umadevi and Madhavi, 2006), and Egypt (Khalifa et al., 1977). The second intermediate host is various species of freshwater or brackish water fish which belong to the Cyprinidae, Siluridae, and Cobitidae (*Acanthogobius* spp., *Ambassis buruensis*, *Anabas* spp., *Astatotilapia desfontainesi*, *Barbus* spp., *Carrssius* spp., *Cyprinus* spp., *Esomus longimana*, *Gerris filamentosus*, *Glossogobius giurus*, *Hampala macrolepidota*, *Mugil capito*, *Ophicephalus striatus*, *Puntius binotatus*, *Therapon plumbeus*, *Teuthis javus*, and *Tilapia* spp.) (Faust and Nishigori, 1926, Velasquez, 1982, Scholz et al., 1990, Scholz, 1991). Fish-eating birds and mammals, including dogs, cats, and humans, have been reported as the natural definitive hosts (Yamaguti, 1958, Yu and Mott, 1994). Experimental definitive hosts include mice, rats, hamsters, guinea pgs, rabbits, cats, and dogs (Faust and Nishigori, 1926, Chai et al., 2012, Nissen et al., 2013).

In Vietnam, where this parasite is highly endemic, raw or pickled fish, for example, slices of silver carp, sold in Vietnamese restaurants is a major source of infection (Dung et al., 2007). In Thailand and Laos, where fermentation is a traditional preservation method for freshwater fish, semi-fermented fish shortly after preparation is the major source of human infections with liver (*Opisthorchis viverrini*) and intestinal flukes (*H*. *pumilio*, *H*. *taichui*, and *H*. *yokogawai*) (Sukontason et al., 1998). Practically, local fish dishes, namely ‘lab-pla’ and ‘pla-som’ are important sources of infection (Radomyos et al., 1998, Sukontason et al., 1998, Onsurathum et al., 2016). *H*. *pumilio* is distributed in Vietnam, Thailand, Laos, Cambodia, South China, Taiwan, the Philippines, Egypt, Palestine, Iraq, India, Sri Lanka, Bangladesh, and Malaysia (Witenberg, 1929, Pearson and Ow-Yang, 1982, Chai et al., 2005a, Chai et al., 2009a, Chai et al., 2015b).

In Vietnam, the presence of *H*. *pumilio*, *H*. *taichui*, and *H*. *yokogawai*, has been confirmed in human infections in two communes of Nam Dinh Province, a northern part of Vietnam (Dung et al., 2007). The prevalence of small trematode eggs among riparian people in this area was 64.9% (399/615) (Dung et al., 2007). Later, in another commune in Nam Dinh Province, 22.7% (92/405) of household members were positive for small trematode eggs (De and Le, 2011). In Ninh Binh Province, 20.5% (381/1857) of commune people were positive for small trematode eggs (Hung et al., 2015). In Thailand, 12 human cases were first reported by Radomyos et al., 1983, Radomyos et al., 1984 through recovery of adult flukes. Thereafter, northern parts (including Chiang Rai and Chiang Mai Provinces) were found to be endemic areas of this fluke (Radomyos et al., 1998). In Laos, the presence of human *H*. *pumilio* infection was first documented by Chai et al. (2005b) based on recovery of adult flukes after praziquantel treatment and purging in Saravane Province and Vientiane Municipality. Later, in Saravane and Champasak Province, 796 and 247 adult specimens of *H*. *pumilio* were recovered in 5 and 5 patients, respectively (Chai et al., 2013a). In Savannakhet Province, a small proportion of recovered worms from 3 patients (80 among 7693 fluke specimens) were *H*. *pumilio* (Chai et al., 2007). In Luang Prabang Province, total 41 specimens of *H*. *pumilio* were recovered in 4 patients (Sohn et al., 2014). In Xieng Khouang Province, 2268 specimens of *H*. *pumilio* were harvested from 8 egg positive people (Chai et al., 2015b).

In Cambodia, no human cases have been detected until present; however, in 2014, a fish survey in Pursat Province, near the Lake Tonlesap, revealed the infection of freshwater fish with the metacercariae of *H*. *pumilio* (Chai et al., 2014a). In Southern China, the presence of human *H*. *pumilio* infection was documented in Guangdong Province in 1964; however, the background literature was not provided (T. Li et al., 2010). In Taiwan, *H*. *pumilio* was described in earlier times (Nishigori, 1924a, Faust and Nishigori, 1926); however, there have been no reports on human infections with this fluke. In the Philippines, human infections have never been documented. In Egypt, human *H*. *pumilio* infection was first documented by Khalifa et al. (1977) in a 9-year-old child passing diarrheic stools. In Korea, a 5-year old patient was found to be naturally infected with one specimen of *H*. *pumilio*, together with 841 *Gymnophalloides seoi* and *3 Gynaecotyla squatarolae* specimens (Chung et al., 2011).

#### 2.3.3 *Haplorchis yokogawai* (Katsuta, 1932) Chen, 1936

The original description of this species, *Monorchotrema yokogawai*, is based on adult flukes obtained in the small intestine of dogs and cats experimentally fed the metacercariae encysted in the mullet *Mugil cephalus* in Taiwan (Katsuta, 1932b). Its characteristic morphology (Fig. 4) differentiated from other *Haplorchis* species is the size, shape, and number of spines on the ventral sucker (Chen, 1936, Pearson and Ow-Yang, 1982). *H*. *yokogawai* has a small ventral sucker with its apex comprising a large ventral lobe armed with numerous tiny spines (Fig. 4) and a pair of large variable-sized sclerites ventro-dextrally, and 3 small lobes armed with tiny spines (Chen, 1936, Pearson and Ow-Yang, 1982). The total number of tiny spines on the ventral sucker was reported to be 70–74 (Katsuta, 1932b) but difficult to count. Ceca of *H*. *yokogawai* exceed the middle level of the testis (Pearson and Ow-Yang, 1982). *H*. *pumilio* is morphologically similar to *H*. *yokogawai* but the latter has 23–32 I- or Λ-shaped sclerites on the ventral sucker (Pearson and Ow-Yang, 1982). Other species can be differentiated by the presence of 12–16 or 15–21 large spines (*H*. *taichui* or *H*. *parataichui*, respectively), ventral sucker with four lobes and armed dorsal pocket (*H*. *vanissimus*), ventral sucker without lobes but with armed dorsal pocket with tiny spines interrupted mid-ventrally (*H*. *paravanissimus*), or remarkably large ventral sucker (*H*. *wellsi*) (Pearson and Ow-Yang, 1982).

The snail host is *Melanoides* (=* Thiara*) *tuberculata* or *Stenomelania newcombi* (Ito, 1964b, Velasquez, 1982). The second intermediate host is freshwater fish (*Ambassis burunensis*, *Amphacanthus javus*, *Anabas testudineus*, *Boleophthalmus* spp., *Carassius auratus*, *Cirrhinus jullieni*, *Clarias fuscus*, *Cyclocheilichthys* spp., *Gambusia affinis*, *Gerris kapas*, *Hampala dispar*, *Hemiramphus georgii*, *Misgurnus* spp. *Mugil affinis*, *Mullus* spp., *Ophicephalus stratus*, *Pectinirostris* spp., *Puntioplites proctozysron*, *Puntius* spp., and *Tilapia nilotica*) in Taiwan, the Philippines, Laos, Cambodia, Vietnam, South China, Malaysia, northern Thailand, India, Australia, and Egypt (Velasquez, 1982, Rim et al., 2008, Rim et al., 2013, Lobna et al., 2010, Chai et al., 2012, Chai et al., 2014a). Fish-eating birds and mammals, including dogs, cats, cattle, and humans, have been reported as the natural definitive hosts (Yamaguti, 1958, Yu and Mott, 1994). Experimental definitive hosts include mice, cats, dogs, and humans (Katsuta, 1932b).

In Thailand and Laos, consumption of semi-fermented fish shortly after preparation is the major risk for human infections with liver and intestinal flukes, including *H*. *yokogawai* (Sukontason et al., 1998). Notably, local fish dishes, ‘lab-pla’ and ‘pla-som’ are important sources of infection (Radomyos et al., 1998, Sukontason et al., 1998, Onsurathum et al., 2016). In the Philippines, local fish dishes called ‘kinilaw’ (freshwater fish seasoned only with salt and vinegar), ‘sabaw’ (boiling fish for several minutes), and ‘sugba’ (grilling over charcoal) are popularly consumed and seem to be the source of infection with *Haplorchis* spp. (Belizario et al., 2004). In Vietnam, raw or pickeld fish, for example, slices of silver carp, is an example of the infection source (Dung et al., 2007). *H*. *yokogawai* is now known to distribute in Thailand, Laos, Cambodia, Malaysia, Vietnam, South China, Taiwan, the Philippines, Hawaii, Egypt, India, Indonesia, and Australia (Velasquez, 1982, Yu and Mott, 1994, Chai et al., 2005a, Chai et al., 2009a, Chai et al., 2014a). Human infections are known in Thailand, Laos, Vietnam, the Philippines, and China.

In Thailand, human infection was first recorded by Manning and Lertprasert (1971), Manning et al. (1971), and then by Kliks and Tantachamrun (1974) and Radomyos et al. (1984). Subsequently, northern parts of Thailand (including Chiang Rai and Chiang Mai Provinces) were found to have human cases infected with *H*. *yokogawai* (Radomyos et al., 1998). In another study performed in Phrae Province, the prevalence of *H*. *yokogawai* among 87 worm-recovery cases was 2.3%, much lower than 64.4% of *H*. *taichui* (Pungpak et al., 1998). In Laos, the presence of human infection was first documented by Chai et al. (2005a) based on adult flukes recovered after praziquantel treatment and purging. Low grade infections were subsequently detected in Savannakhet Province (Chai et al., 2007). In another study in Savannakhet and Saravane Province, adult specimens were recovered from a small number of patients (Sayasone et al., 2009). In Phongsali Province, *Haplorchis* spp., predominantly *H*. *taichui* and a small number of *H*. *yokogawai* were recovered from local people (Chai et al., 2010). Similarly, in Luang Prabang Province, adults were recovered in 5 of 10 patients treated with praziquantel (Sohn et al., 2014). In Vietnam, the presence of *H*. *yokogawai*, together with *H*. *pumilio*, *H*. *taichui*, and other intestinal flukes, has been first confirmed in human infections in two communes of Nam Dinh Province (Dung et al., 2007).

In the Philippines, human infections were described by Africa et al. (1940) from 16 of 33 human autopsies. Later, however, the presence of this parasite has seldom been documented. In Southern China, the presence of human *H*. *pumilio* infection was first documented in Guangdong Province in 1979; however, the literature background was not provided (T. Li et al., 2010). In Taiwan where *H*. *yokogawai* was originally discovered, the possibility of human infection was proved by a human experimental infection (Katsuta, 1932b); however, no reports are available on natural human infections. In Cambodia, a recent fish survey in Pursat Province, near the Lake Tonlesap, revealed the infection of freshwater fish (*Puntioplites falcifer*) with the metacercariae of *H*. *yokogawai* (Chai et al., 2014a); however, no human infection cases have been detected.

#### 2.3.4 *Haplorchis vanissimus* (Africa, 1938) Yamaguti, 1958

The original description of this species is based on adult flukes recovered at an autopsy of a Filipino (Africa, 1938). It was morphologically redescribed by Pearson (1964) in Australia. Its specific morphology includes the presence of four armed lobes and an armed dorsal pocket on the ventral sucker with spines continuous across the width of ventral face (Pearson, 1964, Pearson and Ow-Yang, 1982). It is readily distinguished from other species of *Haplorchis* (*H*. *pumilio*, *H*. *parapumilio*, *H*. *taichui*, *H*. *parataichui*, *H*. *yokogawai*, *H*. *wellsi*, and *H*. *sprenti*) by the form and spination of the ventral sucker (Pearson, 1964, Pearson and Ow-Yang, 1982). The most closely resembled species is *H*. *paravanissimus* which has a simple ventral sucker without obvious lobes and the presence of minute spines interrupted mid-ventrally (Pearson and Ow-Yang, 1982).

The snail host has not been confirmed. Freshwater fish probably play the role of the second intermediate hosts (Yu and Mott, 1994). In Australia, fish-eating birds were found to be natural definitive hosts (Pearson, 1964). No further human infections have been documented. The practical mode of human infection or the type of fish dish involved is unknown. The geographical distribution of this fluke is confined to the Philippines and Australia.

### 2.4 Pygidiopsis

#### 2.4.1 *Pygidiopsis summa* Onji and Nishio, 1916

The original description of this species is based on adult flukes recovered from dogs fed brackish water fish infected with the metacercariae in Japan (Onji and Nishio, 1916). Its characteristic morphologies include a small concave body, median location of the ventral sucker, unique morphology of the ventrogenital apparatus (having two groups of spines on the gonotyl; 5–6 right side and 7–9 left side), side-by-side location of the two testes (Chai et al., 1986a), and small pyriform eggs with no distinct muskmelon patterns on the shell (Lee et al., 1984a). In *P*. *genata*, the ventral sucker is median and globular in shape, whereas in *P*. *summa*, it is slightly submedian and transversely elliptical; the former has only one group of spines on the gonotyl whereas the latter has two groups of spines (Chai et al., 1986a).

The life cycle of *P*. *summa* has been elucidated by Ochi (1931) in Yamaguti, Hiroshima, and Okayama Prefectures, Japan. The snail host is the brackish water snail *Tympanotonus microptera* (=* Cerithidea fluviatilis*) in Korea (Chai, unpublished observation) and in Japan (Ochi, 1931). The metacercariae are detected in the gill and muscle of the mullets (*Mugil cephalus* and *Liza menada*), redlip mullets (*Chelon haematocheilus*), and gobies (*Acanthogobius flavimanus*) (Onji and Nishio, 1916, Chun, 1963, Sohn et al., 1994a, Kim et al., 2006, Sohn, 2009, Cho et al., 2012). Natural infections of domestic or feral cats (Eom et al., 1985b, Sohn and Chai, 2005, Shin et al., 2015) and raccoon dogs (E.H. Shin et al., 2009) have been reported. Experimental hosts include rats, cats, and dogs (Ochi, 1931, Chun, 1963).

Humans are contracted by this fluke through consumption of raw or undercooked brackish water fish, in particular, the mullet, goby, and perch. It is now known to distribute in Japan, Korea, and Vietnam (Yokogawa et al., 1965, Seo et al., 1981b, Vo et al., 2008, Sohn et al., 2016) (Table 4). Human infections with *P*. *summa* were first reported by detection of eggs in human feces in Japan (Takahashi, 1929b). Subsequently, adult flukes were identified from humans in Japan (Yokogawa et al., 1965) and Korea (Seo et al., 1981b). In Korea, eight people residing in a salt-farm village of Okku-gun, Jeollabuk-do who habitually ate the raw flesh of the mullet were proved to be infected with *P*. *summa* through recovery of adult flukes after bithionol treatment and purging (Seo et al., 1981b). In another coastal area of South Korea, 18 heterophyid egg-positive people were found to be mixed-infected with *P*. *summa* and *Heterophyes nocens* (Chai et al., 1994b). Eighteen more cases were detected in another coastal area, Muan-gun, Jeollanam-do, from whom total 703 adult specimens of *P*. *summa* were recovered (Chai et al., 1997). Five more cases were detected in Buan-gun, Jeollabuk-do, and total 1732 adult specimens were collected (Chai et al., 1998). Subsequently, more wide distribution of this parasite has been found along the western and southern coastal islands of South Korea, although the egg positive rate was remarkably higher for *Heterophyes nocens* (11.0%) than that for *P*. *summa* (1.2%) (Chai et al., 2004).

**Table 4:** Other heterophyid species infecting humans around the world.

Species	Human infection	Country/region	First reporter
*Pygidiopsis summa*	Yes	Korea, Japan, Vietnam	Onji and Nishio (1916)
*Pygidiopsis genata*	Yes	Egypt, Palestine, Rumania, Iran, Ukraine, Tunisia, Israel, Kuwait, Philippines	Looss (1907)
*Heterophyopsis continua*	Yes	Korea, Japan, China, Vietnam, Saudi Arabia, United Arab Emirate	Onji and Nishio (1916)
*Stellantchasmus falcatus*	Yes	Korea, Japan, Philippines, USA (Hawaii), Thailand, Vietnam, China, Taiwan, Laos, Australia, India, Iran, Palestine	Onji and Nishio (1916)
*Centrocestus formosanus*	Yes	Taiwan, China, Japan, Philippines, India, Vietnam, Laos, Thailand, Turkey, Croatia, USA, Mexico, Costa Rica, Colombia, Brazil	Nishigori (1924b)
*Centrocestus armatus*	Ye	Japan, Korea	Tanabe (1922)
*Centrocestus cuspidatus*	Yes	Egypt, Taiwan, China, Kuwait, Tunisia	Looss in 1896 (Looss, 1902)
*Centrocestus kurokawai*	Yes	Japan	Kurokawa (1935)
*Stictodora fuscata*	Yes	Korea, Japan, Kuwait	Onji and Nishio (1916)
*Stictodora lari*	Yes	Korea, Japan, Australia, Russia, Vietnam	Yamaguti (1939)
*Procerovum varium*	Yes	Japan, China, Philippines, Cambodia, Laos, Vietnam, Australia, India, Korea	Onji and Nishio (1916)
*Procerovum calderoni*	Yes	Philippines, China, Egypt	Africa and Garcia (1935)
*Acanthotrema felis*	Yes	Korea	Sohn et al., 2003a, Sohn et al., 2003b
*Apophallus donicus*	Yes	USA, Canada, Europe	Skrjabin and Lindtrop in 1919 (Price, 1931)
*Ascocotyle longa*	Yes	USA, Brazil, Mexico, Colombia, Panama, Peru, Czech Republic, Germany, Greece, Romania, Egypt, Georgia, Israel, Turkey	Ransom (1920)
*Cryptocotyle lingua*	Yes	UK, Denmark, Iceland, Norway, Russia, Russia, North America, Japan, Greenland	Creplin in 1825 (Stunkard, 1930)

#### 2.4.2 *Pygidiopsis genata* Onji and Nishio, 1916

The original description of this species is based on adult flukes recovered from the small intestine of a pelican in Cairo, Egypt (Looss, 1907, Ransom, 1920). Its characteristic morphologies include the presence of 16 small spines on the oral sucker which are seen only in fresh preparations, pear-shape or triangular body, short ceca terminating at the level of the ovary, and a small oval gonotyl on the ventrogenital sac (Witenberg, 1929). In *P*. *summa*, the body is ovoid to oval, with relatively long ceca terminating near the anterior border of testes, and two small oval gonotyl armed with spines (Chai et al., 1986a).

The snail host is *Melanoides tuberculata* (Youssef et al., 1987b) and *Melanopsis costata* (Dzikowski et al., 2004). The second intermediate host is brackish water fish (including *Barbus canis*, *Mugil capito*, and *Tilapia* spp.) (Witenberg, 1929, Mahdy and Shaheed, 2001, Ibrahim and Soliman, 2010, Lobna et al., 2010), and experimentally *Gambusia affinis* and *Tilapia nilotica* (Youssef et al., 1987b). Natural definitive hosts include wolves, cats, dogs, foxes, shrews, rats, pelicans, kites, ducks, and cormorants (Looss, 1907, Witenberg, 1929, Kuntz and Chandler, 1956, Dzikowski et al., 2004). Experimental hosts include mice, rats, and hamsters (Mansour et al., 1981).

The mode of human infection is probably the consumption of raw or undercooked brackish water fish. Human infections were unknown before 1987 when eggs were detected in human fecal examinations from Maryut area on Manzala Lake, Egypt; the egg positive rate was 2.7% (2/73) (Youssef et al., 1987a). Although the adult flukes were not identified, the eggs of *P*. *genata* were morphologically well discriminated from those of *Heterophyes heterophyes* (Youssef et al., 1987a). The existence of human infections in other countries, including Rumania, Iran, Palestine, Ukraine, Tunisia, Israel, and Kuwait should be determined. After its discovery, *P*. *genata* was found in pelicans in Romania (Ciurea, 1924), dogs in China (it should be verified whether the species was actually *P*. *genata* or a related species *P*. *summa*) (Faust and Nishigori, 1926), and dogs and cats (both hosts were experimentally infected) in the Philippines (Africa and Garcia, 1935, Africa et al., 1940), and a Persian wolf, dogs, and cats in Palestine (Witenberg, 1929). In Egypt, this parasite has been found from cats, dogs, foxes, shrews, rats, kites, and ducks (Kuntz and Chandler, 1956). Recently, this parasite was recovered from birds in Israel (Dzikowski et al., 2004) and stray cats in Kuwait (El-Azazy et al., 2015).

### 2.5 Heterophyopsis

#### 2.5.1 *Heterophyopsis continua* (Onji and Nishio, 1916) Price, 1940

The original description of this parasite is based on specimens recovered from experimental cats fed the mullet harboring the metacercariae in Japan (described under the name *Heterophyes continuus*) (Onji and Nishio, 1916). Tubangui and Africa (1938) created a new genus *Heterophyopsis*. Yamaguti (1939) also created a new genus *Pseudoheterophyes* and assigned *H*. *continuus* to this genus. However, Africa et al. (1940) and Price (1940) synonymized *Pseudoheterophyes* with *Heterophyopsis* and thereafter, the status of the genus *Heterophyopsis* has been settled. Its characteristic morphology includes the elongate body, genital sucker located separately at a slightly postero-sinistral position from the ventral sucker, and two obliquely tandem testes (Seo et al., 1984a, Chai and Lee, 2002). The most similar genus *Heterophyes* is different from *Heterophyopsis* in having a genital sucker closely adjacent with the ventral sucker, whereas the genital sucker is much separated from the ventral sucker; the former has a ovoid to elliptical or pyriform body but the latter has an elongated body (Chai, 2007).

The first intermediate host is yet unknown. Metacercariae encyst in the perch *Lateolabrax japonicas*, goby *Acanthogobius flavimanus*, *Konosirus* (=* Clupanodon*) *punctatus*, and *Conger myriaster* (Chun, 1960b, Seo et al., 1984a, Sohn et al., 1994a, Sohn et al., 2009a, Chai et al., 2005a, Kim et al., 2006, Cho et al., 2012). Metacercariae were also detected in the sweetfish (*P*. *altivelis*), gobies (*Boleophthalmus pectinirostris* and *Scartelaos* sp.), mullets (*M*. *cephalus*), and groupers (*Epinephelus bleekeri* and *E*. *coioides*) (Sohn et al., 2005, Kim et al., 2006, Vo et al., 2008, Vo et al., 2011, Thanh et al., 2009, Chai et al., 2009a). Natural definitive hosts are domestic or feral cats (Eom et al., 1985b, Sohn and Chai, 2005, Schuster et al., 2009, Chai et al., 2013c), dogs (Yamaguti, 1958), ducks (Onji and Nishio, 1916), and sea-gulls (Yamaguti, 1939). Experimental definitive hosts include cats (Onji and Nishio, 1916), dogs (Chun, 1960b, Seo et al., 1984a) and domestic chicks (Hong et al., 1990, Shin et al., 2015).

The presence of human infections was mentioned in Japan (Yamaguti, 1958, Komiya and Suzuki, 1966); however, no proper literature background is available for this. Subsequently, in Korea, two natural human infections were first discovered (Seo et al., 1984a). To date, total 17 human cases, including the first two cases, have been confirmed by recovery of adult flukes in Korea (Hong et al., 1996, Guk et al., 2006, Park et al., 2007, Chai et al., 1997, Chai et al., 1998, Chai et al., 2004, Chai et al., 2014a, Chai et al., 2015b). It is now distributed in Korea (Seo et al., 1984a, Chai et al., 2009b), China (Yu and Mott, 1994), Japan (Yamaguti, 1958, Komiya and Suzuki, 1966), Vietnam (Vo et al., 2008, Chai et al., 2012), Saudi Arabia (under the name *Heterophyopsis* sp.; Kalantan et al., 2000), and United Arab Emirates (Schuster et al., 2009).

### 2.6 Stellantchasmus

#### 2.6.1 *Stellantchasmus falcatus* Onji and Nishio, 1916

The original description of this species was based on adult flukes obtained from cats, dogs, and a bird experimentally fed the mullet harboring the metacercariae in Japan (Onji and Nishio, 1916). Witenberg (1929) created a new genus *Diorchitrema* for the flukes obtained from the intestine of dogs and cats from Palestine, reporting them as *D*. *pseudocirrata*. However, Alicata and Schattenberg (1938) and Price (1940) synonymized *Diorchitrema* with *Stellantchasmus*, placing *D*. *pseudocirrata* as a synonym of *S*. *falcatus*. In Taiwan, *Stellantchasmus formosanus* and *Stellantchamus amplicaecalis* were reported as new species; however, both species were synonymized with *S*. *falcatus* (Pearson, 1964). The adult flukes are morphologically characterized by its pyriform shape with the presence of a small submedian ventral sucker and an expulsor type elongated sac-like seminal vesicle (Seo et al., 1984b, Chai and Lee, 2002).

The snail host has been confirmed to be the brackish water snail (*Melanoides tuberculata*, *Stenomelania newcombi*, *Thiara granifera*, and *T*. *granifera mauiensis*) (Martin, 1958, Noda, 1959). The second intermediate host was shown to be brackish water fish, in particular, the mullets (*M*. *cephalus*, other *Mugil spp*., *Liza menada*, and L. *haematocheila*) and gobies (*Acanthogobius flavimanus*) (Alicata and Schattenburg, 1938, Martin, 1958, Chai and Sohn, 1988, Chai et al., 2009a). However, freshwater fish, in particular, the half-beaked fish (*Dermogenus pusillus*) and climbing perch (*Anabas testudineus*) also serve as the second intermediate host in the northern part of Thailand (Wongsawad et al., 2004, Wongsawad and Wongsawad, 2010). Analysis of the genomic DNA revealed that the two types (the freshwater type and the brackish water type) of *S*. *falcatus* are strongly suggested to be different species (Wongsawad and Wongsawad, 2010). Further stdies are warranted to verify this point. Natural definitive hosts are stray cats, dogs, pigs, rats, humans, and birds (Takahashi, 1929b, Seo et al., 1984b, Sohn and Chai, 2005, Anh et al., 2009, Shin et al., 2015). Experimental hosts include chicks, mice, rats, cats, dogs, and humans (Katsuta, 1931, Alicata and Schattenburg, 1938, Wongsawad et al., 1998, Saenphet et al., 2003, Ramsuk et al., 2004).

Human infections with *S*. *falcatus* were first detected by recovery of eggs in human feces (105 of 6680 inhabitants) in Japan, but the adult flukes were not confirmed (Takahashi, 1929b). Katsuta (1931) succeeded in infecting himself with *S*. *formosanus* (a synonym of *S*. *falcatus*), and Africa and Garcia (1935) detected people infected with *S*. *falcatus* (under the name *D*. *pseudocirrata*) by adult worm recovery in the Philippines. Thereafter, a few human cases were found by recovery of adult flukes in Hawaii (Alicata and Schattenburg, 1938, Glover and Alicata, 1957), Japan (Kagei et al., 1964, Ito, 1964a), Thailand (Kliks and Tantachamrun, 1974, Tantachamrun and Kliks, 1978, Radomyos et al., 1990), Korea (Seo et al., 1984b, Hong et al., 1986, Chai et al., 1998), and Vietnam (Dung et al., 2007). Now this fluke is known to distribute in Japan, the Philippines, Hawaii, Korea, China, Taiwan, Laos, Vietnam, Thailand, Cambodia, Australia, India, Iran, and Palestine (Pearson, 1964, Ditrich et al., 1990, Radomyos et al., 1990, Wongsawad et al., 1997, Wongsawad et al., 1998, Farahnak et al., 2004, Chai et al., 2009a, Chai et al., 2016).

### 2.7 Centrocestus

#### 2.7.1 *Centrocestus formosanus* (Nishigori, 1924) Price, 1932

The original description of this species was based on worms obtained from experimental dogs, cats, mice, rats, and ducks fed freshwater fish containing the metacercariae and found also in a naturally infected night herons (*Nycticorax nycticorax*) in Taiwan (Nishigori, 1924a, Nishigori, 1924b). Thereafter, numerous species of this genus have been described. However, Chai et al. (2013a) proposed the recognition of only six species, namely, *C*. *armatus*, *C*. *cuspidatus*, *C*. *formosanus*, *C*. *kurokawai*, *C*. *polyspinosus*, and *C*. *asadai* in the genus *Centrocestus*. The adult flukes are morphologically characterized by having a body rounded posteriorly and tapering anteriorly, no caudal appendage of the oral sucker, and 30–36 circumoral spines around the oral sucker (Witenberg, 1929, Chen, 1942). The number of oral spines is the most useful key to differentiate the species; *C*. *armatus* has 42–48 circumoral spines, *C*. *cuspidatus* 36 spines, *C*. *kurokawai* 38–40 spines, *C*. *polyspinosus* 50–60 spines, and *C*. *asadai* 26–30 spines (Waikagul et al., 1997, Chai, 2007).

The snail host is *S*. *libertina* in Taiwan (Nishigori, 1924a, Nishigori, 1924b, Ito, 1964b), *Stenomelania newcombi* in Hawaii (Martin, 1958), and *Melanoides tuberculata* in Taiwan (Lo and Lee, 1996), USA, and Mexico (Scholz and Salgado-Maldonado, 1999, Mitchell et al., 2000). Various species of fish, including *Anabas testudineus*, *Astyanax fasciatus*, *Carassius auratus*, *Cirrhinus molitorella*, *Cyclocheilichthys armatus*, *C*. *repasson*, *Cyprinus carpio*, *Etheostoma fonticola*, *Gambusia affinis*, *Hampala dispar*, *Hypsibarbus pierrei*, *Misgurnus anguillicaudatus*, *Mystacoeleucus atridorsalis*, *M*. *greenwayi*, *Ophicephalus striatus*, *Oreochromis aureus*, *Osteochilus hasselti*, *Physoschistura meridionalis*, *P*. *parva*, *Puntioplites proctozysron*, *Puntius brevis*, *P*. *gonionotus*, *P*. *leiacanthus*, *Tilapia nilotica*, *T*. *zillii*, and *Zacco platypus*, serve as the second intermediate host (Chen, 1942, Martin, 1958, Waikagul et al., 1997, Srisawangwong et al., 1997, Mitchell et al., 2000, Han et al., 2008, Rim et al., 2008, Rim et al., 2013). Natural definitive hosts are fish-eating birds and mammals, such as the green heron (*Butorides vitrescens*), heron (*Butorides striatus*), pond heron (*Ardeola grayii*), night heron (*N*. *nycticorax*), great egret (*Ardea alba*), and dog (Chen, 1942, Premvati and Pande, 1974, Scholz and Salgado-Maldonado, 1999, Mitchell et al., 2000). Experimental definitive hosts include mice, rats, dogs, cats, rabbits, and ducks (Nishigori, 1924a, Nishigori, 1924b, Chen, 1942, Waikagul et al., 1997, Saenphet et al., 2006a, Han et al., 2008, Pinto et al., 2015).

Regarding human infections, an experimental infection was performed successfully when this species was first reported as a new species (Nishigori, 1924a, Nishigori, 1924b). After then, possible occurrence of natural human infections was suggested by several authors (Ito, 1964a, Premvati and Pande, 1974). T. Li et al. (2010) listed *C*. *formosanus* as one of the human infecting helminths found in 1979 in Guangdong Province, China, but there is no related literature provided. Yu and Mott (1994) described the presence of human *C*. *formosanus* infections in China, Taiwan, and the Philippines without proper literatures. It is unclear whether these reports were truly based on identification of adult worms. Waikagul et al. (1997) reported two human cases infected with *C*. *formosanus* (under the name *C*. *caninus*) in Thailand. This can be regarded as the first adult-proven human *C*. *formosanus* infections. The next report on natural human *C*. *formosanus* infections (3 cases) was published from Vietnam (De and Le, 2011). Although there are no detailed worm descriptions, the figures of the whole worm and of the 32 circumoral spines on the oral sucker apparently indicate that the worms are *C*. *formosanus* (De and Le, 2011). Thereafter, ten additional human cases were reported from Lao PDR by recovery of adult flukes after chemotherapy and purging (Chai et al., 2013a, Chai et al., 2013b, Chai et al., 2015a). This species is now known to distribute in Taiwan, China, Japan, the Philippines, India, Vietnam, Lao PDR, Thailand, Turkey, Croatia, USA (Hawaii, Florida, Texas, and Utah), Mexico, Colombia, and Brazil (Chen, 1942, Martin, 1958, Yamaguti, 1975, Yu and Mott, 1994, Waikagul et al., 1997, Scholz and Salgado-Maldonado, 1999, Mitchell et al., 2000, Velásquez et al., 2006, Gjurčevic et al., 2007, De and Le, 2011, Pinto et al., 2013, Chai et al., 2013a, Chai et al., 2013b). Interestingly, *C*. *formosanus* became an exotic trematode species in the New World (USA, Mexico, Costa Rica, and Brazil) that originated from Southeast Asia from the late 1950s (Scholz and Salgado-Maldonado, 1999, Mitchell et al., 2000, Cortés et al., 2010, Pinto and de Melo, 2010, Johnson et al., 2012). Human infections are unknown in USA, Mexico, and Brazil (Chai et al., 2013a).

#### 2.7.2 *Centrocestus armatus* (Tanabe, 1922) Price, 1932

The original description of this species was based on worms recovered from dogs, cats, rabbits, rats, and mice experimentally fed cyprinoid fish harboring the metacercariae in Japan (Tanabe, 1922). Morphological characteristics of *C*. *armatus* include the presence of 42–48 circumoral spines on the oral sucker, small number of intrauterine eggs, median location of the ovary, and side-by-side location of two testes (Hong et al., 1988, Chai, 2007). Regarding human infections, a successful experimental infection was reported in Japan when this fluke was first reported as a new species (Tanabe, 1922). Later, a naturally infected human case was reported in Korea (Hong et al., 1988). To date, the geographical distribution of this species is limited to Korea and Japan.

The first intermediate host is the fresh water snail, *S*. *libertina* (Takahashi, 1929c, Komatsu et al., 2014). The second intermediate hosts are various species of freshwater fish, such as, *Zacco platypus*, *Nipponocypris temminckii* (=* Zacco temminckii*), *Rhodeus ocellatus*, *Gobius similis*, *P*. *parva*, and *Pelteobagrus fulvidraco* (Chai et al., 2009a, Komatsu et al., 2014). The large egret *Egretta alba modesta* (Ryang et al., 1991), kites, herons, and three unidentified avian species (Komatsu et al., 2014), and the cat (Sohn and Chai, 2005, Shin et al., 2015) have been reported to be the natural definitive hosts. Rats and hamsters were useful experimental definitive hosts that allowed full development of worms than rats, mice, and chicks (Hong et al., 1989, Kimura et al., 2007).

#### 2.7.3 *Centrocestus cuspidatus* (Looss, 1896) Looss, 1899

The original description of this species was based on adult flukes recovered from a naturally infected dog in Egypt (Ransom, 1920). The adult fluke has 36 circumoral spines (Ransom, 1920), which is a unique feature differed from other *Centrocestus* spp. The snail intermediate host is yet to be determined. The second intermediate host is freshwater fish, including *Gambusia* sp. (Yamaguti 1975). The reservoir hosts include dogs, cats, foxes, rats, and chickens (Yu and Mott, 1994, El-Azazy et al., 2015). Adult flukes were obtained experimentally from rats fed the fish *Astatotilapia desfontainesi* (Yamaguti, 1958) and chicks fed the metacercariae from *Gambusia* (Yamaguti, 1975). Human infections were found in Egypt, Taiwan, and mainland China (Yu and Mott, 1994, Li et al., 2010); however, the precise literature background is unavailable for this. Now, this fluke is known to distribute in Egypt (Ransom, 1920, Martin, 1959), Kuwait (El-Azazy et al., 2015), Tunisia (Yamaguti, 1958), Taiwan (Yu and Mott 1994), and China (Li et al., 2010, Li et al., 2010).

#### 2.7.4 *Centrocestus kurokawai* (Kurokawa, 1935) Yamaguti, 1958

The original description of this species was based on worms recovered from a naturally infected human in Hiroshima Prefecture, Japan (under the name *C*. *formosanus* var. *kurokawai*) (Kurokawa, 1935). The adult worm has 38–40 circumoral spines (Kurokawa, 1935), and it was treated as a distinct species by Yamaguti (1958). Ito (1964a) and Yu and Mott (1994) continued to use *C*. *formosanus* var. *kurokawai* or *C*. *kurokawai* for this species. On the other hand, Waikagul et al. (1997) synonymized them and used the name *C*. *armatus* for *C*. *kurokawai*. However, Chai et al. (2013b) retained the name *C*. *kurokawai* in consideration that the number of circumoral spines (38–40) may be a specific feature. No information is available on the intermediate hosts and reservoir hosts. The second intermediate host may be some species of freshwater fish (Yu and Mott, 1994).

### 2.8 Stictodora

#### 2.8.1 *Stictodora fuscata* (Onji and Nishio, 1916) Yamaguti, 1958

The original description of this species was based on specimens recovered from cats experimentally fed infected mullets in Japan (Onji and Nishio, 1916). Its characteristic morphology includes the presence of a gonotyl superimposed on the ventral sucker and armed with 12 chitinous spines, a metraterm, and two testes located obliquely in the middle field of the body (Chai et al., 1988). The snail intermediate host is not yet determined (Komiya, 1965, Chai et al., 2009a). The second intermediate host is brackish water fish, including mullets (*M*. *cephalus* and *Liza macrolepis*), redlip mullets (*Chelon haematocheilus*), and gobies (*A*. *flavimanus*) (Onji and Nishio, 1916, Sohn et al., 1994a, Sohn et al., 1994b, Abdul-Salam et al., 2000, Cho et al., 2012). Natural definitive hosts include cats, dogs, humans (Onji and Nishio, 1916, Yamaguti, 1958, Sohn and Chai, 2005, Chai et al., 2013c), and birds (Komiya, 1965, Yamaguti, 1971). Cats can be an experimental definitive host (Onji and Nishio, 1916, Sohn et al., 1994b, Abdul-Salam et al., 2000).

The presence of human infection with this fluke was briefly mentioned by Yamaguti, 1958, Yamaguti, 1971 but without literature background. Later, a human infection was discovered from a Korean young man (under the name *Stictodora* sp.), who enjoyed eating raw mullets and gobies (Chai et al., 1988). Thereafter, about 35 human infection cases have been detected by recovery of adult flukes in seashore villages in Korea (Chai et al., 1994b, Chai et al., 1997, Chai et al., 1998, Chai et al., 2004, Chai et al., 2014c, Chai et al., 2015a, Guk et al., 2006, Park et al., 2007). The existence of *S*. *fuscata* life cycle has been reported in Korea (Chai et al., 2009a), Japan (Komiya, 1965), and Kuwait (Abdul-Salam et al., 2000).

#### 2.8.2 *Stictodora lari* Yamaguti, 1939

The original description of this fluke was based on worms discovered in the small intestine of the sea gull *Larus crassirostris* in Japan (Yamaguti, 1939). Its morphological characters include a gonotyl armed with 70–80 minute spines arranged in two groups; one densely crowded group of 30–40 spines and the other linearly-arranged group containing 30–40 spines, which make a C-form or a comma- or reversed comma-shape (Chai et al., 1989a). The first intermediate host is the brackish water gastropod (*Velacumantus australis*) (Bearup, 1961). A number of estuarine fish species (including *Flavingobius lateralis obliquus*, *Atherinosoma microstoma*, *Urocampus carinorostris*, *Waiteopsis paludis*, *Mugil* sp., and *Gambusia affinis*) have been identified as the fish hosts (Bearup, 1961). Natural definitive hosts include feral cats (Sohn and Chai, 2005, Chai et al., 2013c, Shin et al., 2015), humans (Chai et al., 2002), and dogs (Anh et al., 2009). Cats, dogs, and seagulls were used as experimental definitive hosts (Belogurov et al., 1968, Bearup, 1961, Chai et al., 1989a, Chai et al., 1989b).

*S*. *lari* has been found in Australia (Bearup, 1961), Russia (Belogurov et al., 1968), Korea (Chai et al., 1989a), and Vietnam (Anh et al., 2009). Human infections were first documented by Chai et al. (2002b) in Korea; 6 human patients were found infected with 1–10 specimens of *S*. *lari* in southern coastal areas. Subsequently, five additional cases were detected in Korea (Chai et al., 2004, Cho et al., 2010).

### 2.9 Procerovum

#### 2.9.1 *Procerovum varium* Onji and Nishio, 1916

The original description of this parasite was based on adult flukes recovered from experimental dogs infected with the metacercariae encysted in the mullet *Mugil cephalus* in Japan (Onji and Nishio, 1916). It can be discriminated from *Procerovum calderoni* in having a shorter expulsor on the ventrogenital sac (Pearson, 1964). The first intermediate host is the brackish water snail, *Melanoides* (=* Thiara*) *tuberculata* (Umadevi and Madhavi, 2000). The second intermediate hosts are various kinds of freshwater fish (Velasquez, 1973b, Yu and Mott, 1994, Umadevi and Madhavi, 2000, Vo et al., 2008, Thanh et al., 2009, Skov et al., 2009, Chai et al., 2012, Chai et al., 2014a). Natural definitive hosts include cats (Sohn and Chai, 2005, Chai et al., 2013c, Shin et al., 2015) and birds (Umadevi and Madhavi, 2000). Adult flukes were experimentally reared in dogs, chicks, ducklings, and mice (Onji and Nishio, 1916, Umadevi and Madhavi, 2000). Experimental human infections were reported in Japan (Komiya and Suzuki, 1966); however, there have been no reports on natural human infections. It is now known to be distributed in Japan, China, the Philippines, Cambodia, Laos, Vietnam, Australia, India, and Korea (Umadevi and Madhavi, 2000, Sohn and Chai, 2005, Vo et al., 2008, Thanh et al., 2009, Chai et al., 2014a, Eom et al., 2015, Hung et al., 2015).

#### 2.9.2 *Procerovum calderoni* (Africa and Garcia, 1935) Price, 1940

The original description of this species was based on worms recovered in dogs, cats, and two humans in the Philippines (under the name *Monorchotrema calderoni*) (Africa and Garcia, 1935). It was later renamed as *Haplorchis calderoni* (Africa 1938); however, it was subsequently assigned to *Procerovum* Onji and Nishio 1916 by Price (1940). It can be discriminated from *P*. *varium* in that the former has an extensively long expulsor, whereas the latter has a comparatively shorter expulsor (Pearson, 1964). The first intermediate host is the brackish water snail (*Thiara riquetti*) (Velasquez, 1973c). The second intermediate hosts are various kinds of freshwater fish (Velasquez, 1973b, Velasquez, 1973c, Yu and Mott, 1994). Natural reservoir hosts as we all as experimental hosts are dogs and cats (Velasquez, 1973c). This fluke has been reported from the Philippines (Africa and Garcia, 1935, Velasquez, 1973b, Velasquez, 1973c), China (Kobayasi, 1968, Yu and Mott, 1994), and Egypt (Tawfik et al., 2000).

### 2.10 Acanthotrema

#### 2.10.1 *Acanthotrema felis* Sohn, Han and Chai, 2003

The original description of this species was based on worms recovered from the small intestine of stray cats in Korea (Sohn et al., 2003a). Its characteristic morphology includes the presence of a ventrogenital sac armed with three sclerites (two long and pointed and one short and thumblike) (Sohn et al., 2003a). The snail host has not been reported. The metacercariae were discovered in the goby (*Acanthogobius flavimanus*), a brackish water fish species; they were experimentally fed to kittens, and adult flukes were harvested 7 days later (Sohn et al., 2003b). Only cats were reported to be the natural definitive host (Sohn and Chai, 2005, Shin et al., 2015). Human infection was first found in a 70-year-old Korean woman residing in a coastal area of Korea (Cho et al., 2010). Thereafter, four more human infections were detected in Korea (Chai et al., 2014c). There have been no reports on the presence of this fluke in other countries.

### 2.11 Apophallus

#### 2.11.1 *Apophallus donicus* (Skrjabin and Lindtrop, 1919) Price, 1931

The original description of this species was based on specimens recovered from the small intestine of dogs, cats, rats, foxes, and rabbits (experimental) in Europe and North America (Yamaguti, 1958, Yamaguti, 1971). Its morphological details were described by Niemi and Macy (1974). Experimental infection of a human with this species was successful in the United States (USA) (Niemi and Macy, 1974). Other reports of human infections were available in USA (Schell, 1985). Cercariae were shed by the stream snail (*Flumenicola virens*) (Niemi and Macy, 1974). Various kinds of fish, including blackside dace, suckers, squawfish, redside shiners, salmon, and rainbow trout were found naturally infected with the metacercariae (Niemi and Macy, 1974). Reservoir hosts include dogs, cats, rats, foxes, and species of birds (Yamaguti, 1958, Yu and Mott, 1994).

### 2.12 Ascocotyle

#### 2.12.1 *Ascocotyle* (*Phagicola*) *longa* Ransom 1920

The original description of this species was based on specimens from an Alaskan fox from National Zoological Park in Washington DC, USA (Ransom, 1920). The morphological details of this species in relation to other species were given by Scholz (1999). The natural and experimental first intermediate host is the cochliopid snail (*Heleobia australis*) in Brazil (Simões et al., 2010). Freshwater fish, in particular, various species of mullets serve as the second intermediate host (Chieffi et al., 1992, Oliveira et al., 2007, Simões et al., 2010). Various kinds of birds, including the pelican and eagle, and mammals, particularly dogs, are reservoir hosts of this fluke (Chieffi et al., 1992, Simões et al., 2010). Hamsters were an experimental definitive host (Simões et al. 2010). Total 10 cases of human infections presumably due to this species (described as *Phagicola* sp.) were first reported in Brazil (Chieffi et al., 1992). Subsequently, Antunes and Almeida-Dias (1994) added 10 more human patients who consumed raw mullets in previous four months in Brazil. The distribution of this species is now known to be worldwide; North (Mexico and USA) and South America (Brazil, Colombia, Panama, and Peru), Europe (Czech Republic, Germany, Greece, and Romania), Africa (Egypt), and the Middle East (Georgia, Israel, and Turkey) (Scholz, 1999).

### 2.13 Cryptocotyle

#### 2.13.1 *Cryptocotyle lingua* (Creplin, 1825) Fischoeder, 1903

The original description of this species was based on specimens recovered in the intestine of an avian species (*Larus marinus*) (under the name *Distoma lingua* by Creplin in 1825) (Stunkard, 1930). It was renamed as *Cryptocotyle lingua* by Fischoeder in 1903 (Ransom, 1920). Its characteristic morphology includes the presence of a genital sucker connected with the posterior part of the ventral sucker and obliquely located two testes (Ransom, 1920). Cercariae develop in littorina snails (*Littorina littorea*) and metacercariae encyst in freshwater fish (*Gobius ruthensparri* and *Labrus bergylta*) and mullet (*Chelon labrosus*) (Yamaguti, 1958, Matthews and Matthews, 1993). Various fish-eating birds and mammals were reported as reservoir hosts, which included cats, dogs, rats, foxes, gulls, terns, and herons in Europe (including UK, Denmark, Iceland, and Norway), Russia, North America, and Japan (Stunkard, 1930, Yamaguti, 1958, Kristoffersen, 1991, Saeed et al., 2006, Christensen et al., 2015). Adults can be experimentally grown in gulls by feeding metacercarial cysts (Yamaguti, 1958). Human infection with this fluke was reported only one time in Greenland (Babbott et al., 1961). Although adult flukes were not identified, it seemed that the egg size of 40–50 by 24–28 μm (significantly larger than the other members of heterophyids) strongly suggested them to be *C*. *lingua* (Babbott et al., 1961).

## 3 Genomics and molecular studies

Molecular studies using PCR-RFLP (ITS1 and CO1), random amplification of polymorphic DNA (RAPD), and simple sequence repeat anchored PCR (SSR-PCR) have shown that *M*. *yokogawai*, *M*. *takahashii*, and *M*. *miyatai* are genetically distinct from each other (Yu et al., 1997a, Yu et al., 1997b, Yang et al., 2000, Yu and Chai, 2010, Yu and Chai, 2013). Chromosomes and karyotypes were also used to differentiate *M*. *yokogawai*, *M*. *takahashii*, and *M*. *miyatai* (Lee et al., 1999). Gene sequence studies were also performed on 28S ribosomal DNA (rRNA) and CO1 of three *Metagonimus* species, with helpful results (Lee et al., 2004). Nucleotide sequence differences were 23.0% (92/400 bp) between *M*. *miyatai* and *M*. *takahashii*, 16.2% (65/400 bp) between *M*. *miyatai* and *M*. *yokogawai*, and 13.2% (53/400 bp) between *M*. *takahashii* and *M*. *yokogawai* (Lee et al., 2004). In addition, by the neighbor-joining and parsimony methods, *M*. *takahashii* and *M*. *yokogawai* were placed in the same clade, whereas *M*. *miyatai* was placed in a different clade (Lee et al., 2004). This result was agreed by another research group (Thaenkham et al., 2012). In a numerical taxonomy study, interestingly, *M*. *miyatai* was classified as a subspecies level of *M*. *takahashii*, whereas *M*. *yokogawai* and *M*. *takahashii* were distinct taxa (Kim et al., 1991).

Using six species available in Japan, a phylogenetic study was recently performed on the genus *Metagonimus*; *M*. *yokogawai*, *M*. *takahashii*, *M*. *miyatai*, *M*. *hakubaensis*, *M*. *katsuradai*, and *M*. *otsurui* (Pornruseetairatn et al., 2016). It was revealed that the former four species were grouped into one big clade, and the latter two species formed another clade, based on a combined 28S rRNA, ITS2, and *cox*1 sequence dataset (Pornruseetairatn et al., 2016). *M*. *suifunensis* was recently reported as a new species based on molecular analysis of ITS1–5.8S–ITS2 region and 28S nuclear rRNA of adult worms; it formed a separate group from *M*. *yokogawai*, *M*. *takahashii*, *M*. *miyatai*, *M*. *hakubaensis*, *M*. *katsuradai*, and *M*. *otsurui* (Shumenko et al., 2017). No information is available regarding proteomics of *Metagonimus* flukes.

Genomics and molecular characteristics of *Heterophyes* spp. have seldom been the subject of study. Before the study of Masala et al. (2016), the only molecular data available for *Heterophyes* spp. were mitochondrial cytochrome *c* oxidase 1 (CO1) and nuclear ribosomal gene (28S rRNA) of *H*. *nocens* (Korea) deposited in GenBank with accession numbers AF188119 for CO1 and AF181890 for 28S rRNA. The sequences of internal transcribed spacer 2 (ITS2) and 28S rRNA were recently analyzed in *H*. *heterophyes* (adults from a hamster infected with metacercariae in mullets from Sardinia), *H*. *nocens* (adults from a cat in Korea), *H*. cf. *nocens* (an adult from a hamster infected with metacercariae in mullets from Sardinia, having a smaller number of rodlets on the gonotyl than *H*. *heterophyes*), and *H*. *dispar* (small-sized metacercariae in mullets from Sardinia) (Masala et al., 2016). The results revealed that *H*. *heterophyes* (GenBank no. KU674951 for ITS2 and KU559553 and KU559556 for 28S) and *H*. cf. *nocens* (no. KU674951 for ITS2 and KU559559 for 28S) from Sardinia formed one clade, whereas *H*. *nocens* (no. KU674959 and KU674960 for ITS2) from Korea and *H*. *dispar* (no. KU674953 for ITS2 and KU559560 for 28S rRNA) from Sardinia formed other two distinct clusters (Masala et al., 2016). By this data, it was suggested that *H*. cf. *nocens* may be conspecific with *H*. *heterophyes*, and the number of rodlets on the gonotyl of *H*. *heterophyes* may be wider than previously considered (Masala et al., 2016).

The complete mitochondrial genome of *H*. *taichui* was obtained and comparatively analyzed with other trematodes (Lee et al., 2013). It has been shown that Lao, Thai, and Vietnamese populations of *H*. *taichui* are genetically distant from one another (Thaenkham et al., 2016). In Vietnam, Dung et al. (2013) studied on genetic variations of three Vietnamese isolates; they attributed the low gene flow among the isolates to topographic features that isolated them geographically, including mountainous and wetland areas. However, based on mitochondrial *CO1* gene analysis, another hypothesis was made; Thailand, Laos, and Vietnam population structures of *H*. *taichui* are affected by the national border rather than environmental factors such as common river basins and distribution of intermediate hosts (Thaenkham et al., 2016). Further studies are required to elucidate this point.

The taxonomic relationships of *P*. *summa* with other trematode parasites were studied by Lee et al. (2007). Using 28S rDNA D1 gene analysis, *P*. *summa* was shown to be located in the same clade as *Paragonimus westermani* and the next clade to *M*. *yokogawai* and *M*. *takahashii*, whereas *Clonorchis sinensis* and *Plagiorchis muris* were put in the same clade (Lee et al., 2007). However, analysis of mitochondrial CO1 showed more proximity of *P*. *summa* to *C*. *sinensis* than to *P*. *westermani*, *M*. *yokogawai*, and *M*. *takahashii* (Lee et al., 2007). A numerical taxonomy study reported that *P*. *summa* were more closely related to *Stellantchasmus falcatus* rather than to *Heterophyes* or *Metagonimus* (Kim et al., 1991). A molecular analysis using rRNA ITS1 and ITS2 revealed that *P*. *genata* is genetically more close to *Ascocotyle longa* and *Ascocotyle pindoramensis* than *Metagonimus yokogawai* and *Haplorchis pumilio* (Al-Kandari et al., 2015).

Sequences of nuclear rRNA genes, including 18S rRNA, 28S rRNA, and ITS2 region, were used for analysis of the phylogenetic relationships of *S*. *falcatus* with other heterophyid and opisthorchiid trematodes (Thaenkham et al., 2010, Sripalwit et al., 2015). *S*. *falcatus* was phylogenetically distinct from *Haplorchis* spp., and *Procerovum* spp., and it was indicated that *S*. *falcatus* might have diverged first from a common ancestor of Haplorchiinae species (Thaenkham et al., 2010). *S*. *falcatus* was also far from *Haplorchoides* sp., *Centrocestus* spp., *M*. *yokogawai*, and *O*. *viverrini* (Sripalwit et al., 2015). A numerical taxonomy study reported that *S*. *falcatus* was more closely related to *Pygidiopsis summa* rather than to *Heterophyes* or *Metagonimus* (Kim et al., 1991).

## 4 Pathogenesis and pathology

Two principal factors are generally related to the pathogenicity and virulence of intestinal parasites; they include mechanical and chemical irritations by the flukes (Chai, 2014b). Mechanical irritation is chiefly caused by movement of worms which can give harmful effects to the mucosa (= villous and crypt layers) of the small intestine (Chai, 2014b). Chemical substances produced by the flukes, which include excretory-secretory proteins (ESP), can play a role for not only antigens but also toxins to the host (Chai, 2014b).

The main habitat of adult *Metagonimus* spp. is the mucosa (villi and crypt) of the small intestine, and worms give mechanical and chemical/immunological stimuli to the host intestinal mucosa. In immunocompetent hosts, worms never invade deeper layers of the submucosa, muscularis mucosa, or serosa (Chai, 2015). However, they may invade deeper levels beyond the mucosa in immunocompromised hosts (Chai et al., 1995a, Chai, 2015). Pathological changes in the mucosa lead to difficulty in nutrient absoption from the intestine; increase in permeability in the intestinal mucosa was reported in mice experimentally infected with *M*. *yokogawai* (Ohnishi and Taufen, 1984). The resulting watery diarrhea seems to be due to poor absorption of intestinal secretions from secretory crypt cells (Cho et al., 1985). Decreased enzyme activities were suggested to be associated with malabsorption and diarrhea in acute *M*. *yokogawai* infection (Hong et al., 1991).

Experimental studies on the intestinal histopathology were performed by Chai (1979), Lee et al. (1981), and Kang et al. (1983) using rats, cats, and dogs as the host, respectively. The major histopathological findings were villous atrophy and crypt hyperplasia, with variable degrees of inflammatory reactions (Chai and Lee, 2002, Chai, 2015, Chai, 2017). The infected mucosa showed blunting and fusion of the villi, edema of the villus tips, congestion, goblet cell hyperplasia, mastocytosis, and inflammatory cell infiltrations in the villous stroma, with decreased villus/crypt height ratios (Chai, 1979, Chai et al., 1993b). In a human metagonimiasis patient, a histopathological study of the small intestine was possible, and almost similar intestinal histopathology was observed (Chi et al., 1988). In animals, the intestinal histopathology was normalized at 3–4 weeks after the infection (Chai, 1979, Chai et al., 1995a). In *M*. *miyatai*-infected mice, similar intestinal histopathology was observed; although the degree of mucosal damage was less severe than in *M*. *yokogawai*-infected mice (Yu et al., 1997c).

The intestinal histopathology in *Heterophyes* spp. and *Haplorchis* spp. infection is essentially the same as that seen in *M*. *yokogawai* infection. The adult flukes of *H*. *heterophyes* (Hamdy and Nicola, 1980) or *H*. *nocens* (Ryang et al., 1999) parasitize the middle part of the small intestines; within the crypt of Lieberkuhn in early stages of infection (by day 2–3 post-infection), and the intervillous space in later stages (Chai, 2014b). They can elicit mild inflammatory reactions, together with ulcers, irritation, and superficial necrosis of the site of their attachment to the host intestinal mucosa (Yu and Mott, 1994, Fried et al., 2004, Toledo et al., 2014). In experimental dogs and cats infected with *H*. *heterophyes*, involvement of Peyer's patches and mesenteric lymph nodes by adult flukes were frequently seen (Hamdy and Nicola, 1980). In avian hosts, like sea gulls, the flukes frequently invade extraintestinal or somatic tissues and organs, in particular, the liver, pancreas, and bile duct (Chai, 2014b). Immune responses of the host against the flukes or their ESP may be too strong (hypersensitive) that the host immunity can damage the host itself (Chai, 2014b). The affected mucosa may undergo hypersensitive and allergic reactions, including severe catarrhal inflammation and loss of villi (Chai, 2014b).

In Thailand, a human autopsy case infected with *H*. *taichui* and two cases infected with *H*. *yokogawai* were reported from Udonthani Province (Manning and Lertprasert, 1971, Manning et al., 1971). Another human case was subsequently reported in Chiang Mai, Thailand; worm sections were found deep in the crypt of the ileum (Kliks and Tantachamrun, 1974). Later, three more human cases infected with *H*. *taichui* were reported with evidence of this infection as a pathogenic parasite (Sukontason et al., 2005). Surgical resection of the small intestines revealed the pathological features of mucosal ulceration, mucosal and submucosal hemorrhages, fusion and shortening of villi, chronic inflammation, and fibrosis of the submucosa; *H*. *taichui* worms were found with their mouth-parts attached to the mucosal epithelium and distorting the epithelial lining (Sukontason et al., 2005). Moreover, *H*. *taichui* was suggested to be an etiological agent of irritable bowel syndrome-like symptoms in humans (Watthanakulpanich et al., 2010).

The pathogenicity and pathology as well as mucosal immune responses of the host against *P*. *summa* infection were studied in experimental rats and mice (Seo et al., 1986, Chai et al., 2014b). The mucosal pathology was severe in rodent intestines and characterized by villous atrophy accompanied by crypt hyperplasia and severe stromal inflammation (Seo et al., 1986). However, no deep invasion of worms beyond the submucosal level was found, and the intestinal lesion was restored from three weeks after the infection (Seo et al., 1986). Immune effectors in the intestinal mucosa against *P*. *summa* infection included IEL, goblet cells, mucosal mast cells, and IgA (Chai et al., 2014b).

Six species of heterophyid flukes, including 3 *Haplorchis* spp. (*H*. *taichui*, *H*. *yokogawai*, and *H*. *vanissimus*), *Stellantchasmus falcatus*, *Procerovum calderoni*, and *Carneophallus brevicaeca* (syn. *Heterophyes brevicaeca* Africa and Garcia 1935; *Spelotrema brevicaeca* Tubangui and Africa 1938), were reported to have caused erratic extraintestinal parasitism in humans, which was often fatal (Africa et al., 1940). The most frequently affected site was the heart (valve), brain, and spinal cord, where eggs and adult flukes originating from the small intestinal mucosa (Africa et al., 1940). Yokogawa (1940) suggested that these patients may have been immunocompromised to become susceptible to this type of extraintestinal heterophyid infections.

*Metagonimus* spp. and *Heterophyes* spp. are also highly suggested to be able to cause such erratic parasitism in immunocompromised patients. It is of note that a patient infected with *Metagonimus* sp. underwent intracerebral hemorrhage and diabetes mellitus (Yamada et al., 2008). The eggs of *H*. *heterophyes* (Gallais et al., 1955, Collomb and Bert, 1959, Collomb et al., 1960) and *H*. *nocens* (under the name *H*. *heterophyes*) (Zhang et al., 1990) were found encapsulated in the brain of patients with neurological symptoms. In *H*. *nocens* infection, the eggs were also detected within an intestinal tumor near the appendix in a 10-year old girl in Japan (Nakano and Inuoe, 1955). An experimental background was provided in *H*. *heterophyes* using rats in Egypt; in four of 20 rats, possibly due to immunosuppression as well as malnutrition and malabsorption, the eggs and/or immature worms were found in the intestinal wall, lymph nodes, liver, and spleen which evidently demonstrated the potential to cause extraintestinal spreading of the infection (Elsheikha, 2007). Another histopathological study in *H*. *heterophyes* infection demonstrated deposition of antigens or immune complexes in the kidneys and brain of experimental mice; these deposits were thought to play important roles in histopathological changes in the kidneys and brain (Daoud, 2012).

Despite that *S*. *falcatus* was involved in causing erratic parasitisms in the heart, brain, and spinal cord of humans (Africa et al., 1940), the pathogenicity and pathology as well as mucosal immune responses of the host has seldom been studied. However, innate immunity against *S*. *falcatus* infection was demonstrated in mice and rats; in the former, the worms spontaneously expelled within a week after infection, and in the latter, the worms expelled after 28 days (Saenphet et al., 2003). The blood eosinophils were increased from the first week of infection and remained relatively high until the worms are expelled from these rodent hosts (Saenphet et al., 2003).

## 5 Immunology

Considering that the intestinal histopathology caused by *M*. *yokogawai* was normalized around 3–4 weeks after the infection (Chai, 1979, Chai et al., 1995a), host protective mechanisms (including innate resistance) against worms seem to be significantly operating. The possible immune effectors for the spontaneous recovery include at least 4–5 components. They include intestinal intraepithelial lymphocytes (IELs) (Chai et al., 1994a), lamina propria lymhocytes (LPLs) (Chai et al., 1994a), mucosal mast cells, and goblet cells (Chai et al., 1993b, Chai et al., 2009a). Eosinophils were also increased in the peripheral blood of *M*. *yokogawai*-infected mice (Ohnishi, 1987), although their roles in worm expulsion are unclear. Hong et al. (1993) infected rats with *Neodiplostomum seoulense* (under the name *Fibricola seoulensis*) first and thereafter challenged *M*. *yokogawai* and observed the worm recovery and intestinal histopathology; interestingly, the pre-established *N*. *seoulense* in the duodenum of the rats affected adversely the settlement of *M*. *yokogawai* flukes in the jejunum or ileum.

Immunogold studies revealed that the antigenicity of *M*. *yokogawai* originates from the syncytial tegument, tegumental cell cytoplasms, vitelline cells, and epithelial lamellae of the cecum (Ahn et al., 1991, Rim et al., 1992). SDS-PAGE and immunoblot analyses on crude extracts of metacercariae showed that out of 14 protein bands found 66 kDa and 22 kDa proteins were the parasite-specific antigens (Lee et al., 1993). Han et al. (2014) detected an interesting somatic antigen, 100 kDa in size, from the tegumental layer of *M*. *yokogawai* adults, which commonly reacts against different kinds of trematodes, including *Gymnophalloides seoi* (intestinal fluke), *Paragonimus westermani* (lung fluke), and *Clonorchis sinensis* and *Fasciola hepatica* (liver flukes).

Also, in *Heterophyes* spp. infection, the fact that intestinal histopathology induced is spontaneously restored indicates the development of strong host protective immunity (Toledo et al., 2014). In sera of humans infected with *H*. *heterophyes*, elevated levels of IgG, IgM, and IgE have been detected (El-Ganayni et al., 1989, Martínez-Alonso et al., 1999, Toledo et al., 2014). In the intestine of infected humans, elevated levels of IgG, IgM, and IgA were also reported (El-Ganayni et al., 1989). In *Haplorchis* spp. infection, Sukontason et al. (2001), Kumchoo et al. (2003), and Saenphet et al. (2008) demonstrated spontaneous expulsion of worms from the intestine of mice, chicks, and rats experimentally infected with *H*. *taichui* within 9, 18, and 28 days post infection, respectively, which demonstrated the development of innate and/or acquired immunity of the host. MMCs were significantly increased in number in infected rats peaking at day 21 post infection (Saenphet et al., 2008). Eosinophil counts and serum IgE levels were elevated after infection which peaked at day 14 post infection (Saenphet et al., 2008). In humans infected with *H*. *taichui*, serum antibodies (probably IgG) against *H*. *taichui* were increased as measured by ELISA; enzyme-linked immunoelectron transfer (EITB) assay was useful to discriminate *H*. *taichui* infection (10 kDa band) from *O*. *viverrini* infection (70 kDa band) (Ditrich et al., 1991).

Saenphet et al. (2006b) studied on mucosal mast cell responses in rats experimentally infected with *C*. *formosanus* (under the name *C*. *caninus*); their possible role in worm expulsion was suggested. Pinto et al. (2015) studied on the worm burden, morphology, and fecundity of *C*. *formosanus* in immunosuppressed mice; the worm burden in dexamethasone-treated mice was significantly greater than that in the control (immunocompetent) mice.

## 6 Clinical manifestations

Clinical symptoms due to *Metagonimus* spp. and other heterophyid infections are generally mild and transient, unless the patients are heavily infected, complicated with other diseases, or immunocompromised (Chai and Lee, 2002, Chai et al., 2009a, Yu and Chai, 2013). The most frequent clinical symptoms in *M*. *yokogawai* infection include abdominal pain, diarrhea, lethargy, anorexia, malabsorption, and weight loss (Cho et al., 1984, Seo et al., 1985). The severity of clinical symptoms is closely related to individual worm burdens (Chai, 2015). In immunocompromised individuals, *M*. *yokogawai* infection may cause severe clinical manifestations, possibly including erratic parasitism in vital organs as reported in other heterophyid fluke infections (Africa et al., 1940, Chai, 2007, Yu and Chai, 2013).

Major clinical manifestations in heterophyiasis patients are mild to moderate degrees of abdominal pain, diarrhea, lethargy, anorexia, and weight loss (Chai, 2014b). However, the severity of symptoms may vary and depend on host-side factors such as the intensity of infection, immune status of the patient, and previous history of infection with these flukes (Chai, 2014b). Immunocompromised patients may undergo severer clinical course, including erratic parasitism (= extraintestinal heterophyiasis) in the heart, brain, and spinal cord, as reported by Africa et al. (1940). Both *H*. *heterophyes* and *H*. *nocens* are suspected to be the causes of cerebral involvement, including epilepsy, brain abscess, or brain cyst (Gallais et al., 1955, Collomb and Bert, 1959, Collomb et al., 1960, Zhang and Fan, 1990). In an acute appendicitis case in a 10-year old girl in Japan, *H*. *nocens* eggs were demonstrated within the tumor formed near the appendix (Nakano and Inuoe, 1955).

Few reports regarding the clinical symptoms and signs of human *Haplorchis* spp. infection are available. This is largely because humans in endemic areas are usually mixed-infected with other foodborne trematodes, namely, *O*. *viverrini*, other heterophyids, or lecithodendriids. Thus, the clinical manifestations solely due to *Haplorchis* spp. infection are difficult to assume. Africa et al. (1940) assumed that the symptoms due to *Haplorchis* spp. infection may be intestinal disturbances such as colicky pain and mucous diarrhea, and the degree of such symptoms would depend upon the number of worms, the extent of penetration, or the destruction or necrosis of the epithelium occasioned by the presence of worms. Recently, it has been reported that *H*. *taichui* infection may cause irritable bowel syndrome-like symptoms, and the patients may complain of dyspepsia, nausea, vomiting, lassitude, abdominal pain, flatulence, loose fecal excretion, fever, pallor, abdominal distension, and jaundice with enlarged liver (Watthanakulpanich et al., 2010).

The symptoms and signs due solely to *P*. *summa* infection have not been elucidated. One of the reasons is mixed-infection of the majority of human cases with other heterophyid flukes such as *H*. *nocens* (Chai et al., 1994b, Chai et al., 1997, Chai et al., 1998, Chai et al., 2004). The most heavily infected person ever documented was a 52-year-old Korean man, with 4045 specimens recovered after chemotherapy and purging (Seo et al., 1981b). In this patient, no significant symptoms or signs were presented, and the only abnormal laboratory finding was 7% peripheral blood eosinophilia (Seo et al., 1981b).

## 7 Diagnosis and treatment

Heterophyid fluke infections can be diagnosed by recovery of eggs in fecal examinations (Chai, 2007). A confirmatory diagnosis can be made when adult flukes are detected during gastroduodenoscopy, surgical procedures in the intestine, or at autopsy (Chai, 2007). A tedious but practical way in the field or laboratory is recovery of adult flukes from diarrheic stools following anthelmintic treatment and purging (Chai, 2007).

An important drawback in the diagnosis of *Metagonimus* spp., *Heterophyes* spp., and *Haplorchis* spp. and other heterophyid infection is close similarity of their eggs to other heterophyid species as well as small liver flukes (*Clonorchis sinensis*, *Opisthorchis viverrini*, and *Opisthorchis felineus*) and lecithodendriid flukes (*Prosthodendrium molenkampi* and *Phaneropsolus bonnei*) (Chai and Lee, 2002, Lee et al., 2012, Chai, 2015). Therefore, in areas of mixed infections, specific diagnosis is usually difficult unless the adult flukes are recovered (Chai, 2007). The eggs found in fecal examinations should be expressed as a broad term, i.e., small trematode eggs (STE), minute intestinal fluke eggs (MIF eggs), or at least heterophyid fluke eggs (Lee et al., 1984a, Lee et al., 2012). Potassium permanganate staining was introduced to distinguish the eggs of *H*. *taichui* from those of *O*. *viverrini*; the staining made the muskmelon patterns on the egg shell surface of *O*. *viverrini* more clearly visible (Sukontason et al., 1999).

Among light infection cases with heterophyid flukes (lesss than 100 specimens), there could be false egg negative cases (Chai and Lee, 2002). The number of eggs produced per day per worm (EPDPW) for *M*. *yokogawai* in the human host was reported to be only 14–64 eggs (Seo et al., 1985). The EPDPW of *H*. *taichui* in humans was 82, only slightly higher than that of *M*. *yokogawai* (Sato et al., 2009a). Therefore, the detectability of eggs in feces from such low worm burden cases is negligible (Chai and Lee, 2002). In these cases, serological tests, in particular, ELISA, against *M*. *yokogawai* may be helpful (Chai et al., 1989b, Cho et al., 1987). In *H*. *heterophyes* infection, three immunodiagnostic methods (counter current immunoelectrophoresis, intradermal test, and indirect fluorescent immunoassay) were tried to detect serum antibodies in experimental dogs (Elshazly et al., 2008). However, the diagnosis of erratic parasitism in the heart, brain, or spinal cord is practically impossible unless biopsy or necropsy is performed on the affected lesion (Chai, 2014b).

The specific diagnosis of human *P*. *summa* or *S*. *falcatus* infection is problematic because it is difficult to discriminate these eggs from other heterophyid or opisthorchiid eggs in fecal examinations (Lee et al., 1984a, Lee et al., 2012). However, a favorable point is that *P*. *summa* eggs are very small in size and *S*. *falcatus* eggs are slightly elongated (Lee et al., 1984a, Chai et al., 2004, Lee et al., 2012). The small egg size is also applicable to *P*. *genata* infection in Egypt (Youssef et al., 1987a).

Recently, molecular techniques, including PCR or PCR-RFLP, were introduced to detect heterophyid infections in human feces (Jeon et al., 2012) and food materials (Pyo et al., 2013). The PCR technique could differentiate *M*. *yokogawai* infection from *C*. *sinensis* or *Haplorchis taichui* infection with detection of mixed-infection cases also (Jeon et al., 2012). The PCR targeting 18S rRNA could also detect *M*. *yokogawai* infection in sweetfish and *Gymnophalloides seoi* infection in oysters (Pyo et al., 2013). Thaenkham et al. (2007) developed a rapid and sensitive tool for detecting *H*. *taichui* with low DNA concentrations and for distinguishing *H*. *taichui* from *Opisthorchis viverrini*, *H*. *pumilio*, and *H*. *yokogawai*. They further suggested that PCR-RFLP profiles would be useful for diagnosing mixed *H*. *taichui* and *O*. *viverrini* infection. PCR diagnosis was also applied to detect low-intensity *H*. *taichui* and *O*. *viverrini* infections in field surveys (Lovis et al., 2009). HAT-RAPD technique was developed to discriminate *H*. *taichui* infection from various other infections, including *O*. *viverrini* and *S*. *falcatus* which generated a 256 bp amplicon and showed a positive result only for *H*. *taichui* (Wongsawad et al., 2009). A multiplex PCR assay based on HAT-RAPD results was also developed for detecting *H*. *taichui* (Wongsawad and Wongsawad, 2012). PCR targeting ITS1 and ITS2 regions of ribosomal DNA revealed useful results; particularly in ITS2 region, the amplicon size for *H*. *taichui* was significantly different from that of *O*. *viverrini*, *C*. *sinensis*, and *H*. *pumilio* (Sato et al., 2009b). However, Sato et al. (2010) reported that PCR for copro-DNA targeting ITS1 and ITS2 regions could detect lower number of *H*. *taichui* cases than those with *H*. *taichui* worms expelled after treatment, whereas the same DNA technique was excellent for copro-detection of *O*. *viverrini* infection. Molecular techniques were also used in Vietnam (De and Le, 2011), China (Jeon et al., 2012), and Thailand (Wongsawad et al., 2012, Sato et al., 2015) to detect human infections with *H*. *pumilio* and *H*. *taichui* (Vietnam, China, and Thailand). However, the diagnosis of erratic parasitism in the heart, brain, or spinal cord is difficult unless biopsy or necropsy is done on the affected lesion (Chai, 2014b). Detection of serum antibodies or parasite genetic markers cannot discriminate intestinal versus visceral infections. Cardiac ultrasonography combined with serum antibody and molecular tests would help the diagnosis of possible cardiac infections with heterophyid fluke infections.

The drug of choice for heterophyid fluke infections, including *Metagonimus* spp., *Heterophyes* spp., and *Haplorchis* spp., is praziquantel (Chai, 2007, Chai, 2013). A single oral dose of 10–20 mg/kg is usually satisfactory (Chai, 2013). However, in areas of mixed infection with the liver fluke, a single oral dose of 40 mg/kg praziquantel can be used in mass treatment (Pungpak et al., 1998, Radomyos et al., 1998, Sayasone et al., 2009, Chai et al., 2010, Chai et al., 2013a, Sohn et al., 2014). However, reduced doses, for example, 20–30 mg/kg, were also useful to treat individual patients in endemic areas (Chai et al., 2005a, Chai et al., 2007, Chai et al., 2009b). The cure rate of 95–100% was reported for *M*. *yokogawai* infection (Rim et al., 1978, Lee et al., 1984b), and over 95% cure rate for *H*. *heterophyes* spp. infection (Ata et al., 1988, El-Hawy et al., 1988). Praziquantel is highly safe at this dose even in children and pregnant women (Chai, 2013). Bithionol (Seo et al., 1981b) or niclosamide (El-Hawy et al., 1988, Ramsuk et al., 2004, Kumchoo et al., 2007) can also be used as an alternative drug.

## 8 Prevention and control

The prevention and control measures for heterophyid infections include control of the snail host, control of the fish host, mass chemotherapy of residents in endemic areas, and health education (Chai, 2015). However, snail control and fish control are difficult to perform successfully (Chai, 2015). Mass drug administration (MDA) can temporarily reduce the prevalence and infection intensity (worm load) but reinfection steadily occurs in endemic areas (Chai, 2015). Health education to not consume raw or undercooked fish will help prevention of heterophyid fluke infection; however, the old tradition of enjoying raw fish dish is practically hard to change (Chai, 2014b, Chai, 2015). The infectivity of *M*. *yokogawai* metacercariae in fish can be controlled by gamma-irradiation at 200 Gy (Chai et al., 1995b). However, this method is not feasible in the field due to various reasons, including the necessity of an irradiator, high cost, and low preference of irradiated fish by the consumers (Chai et al., 1995b, Chai, 2015).

The metacercariae of heterophyids can survive only 1 day in pickled fish dish; therefore, consuming ‘pla-som’ after 3 days of preparation would be safe from infection (Sukontason et al., 1998). However, the metacercariae of heterophyids can survive longer than 3 h in ‘lab-pla’ which is usually consumed immediately after preparation; therefore, this type of fish dish may be dangerous for parasite infection (Sukontason et al., 1998). Smoking to a temperature of 65 °C can be considered for parasitic nematodes in fish, but little is known about the effectiveness of this process for intestinal trematodes, including *Metagonimus*, *Heterophyes*, and *Haplorchis* (Chai, 2014b).

## 9 Conclusions

Fishborne zoonotic heterophyid infections are strongly linked to deeply embeded cultural traits, for example, consumption of raw or improperly cooked fish in traditional ways, in each endemic area, which is located mostly in Southeast Asia. Thus, the viscious cycle of reinfection continues in this area, and control effort often results in failure. Nevertheless, national as well as international health policies seldom put foodborne trematode infections, including intestinal heterophyid infections, on high health priorities; thus, these infections have become a long-time neglected group of diseases. In order to provide a better understanding of the global disease prevalence and geographical distribution of these infections, improved diagnostic tools are urgently needed, especially those that can differentiate the various species of parasites involved. Finally, long-term pilot projects with risk assessment studies are needed to provide proper control strategies at local as well as national and international levels.

## Conflict of interest

We do not have any conflict of interest related to this work.
